# Double salt crystal structure of hexa­sodium hemiundeca­hydrogen *α*-hexa­molybdoplatinate(IV) heminona­hydrogen *α*-hexa­molybdoplatinate(IV) nona­cosa­hydrate: di­hydrogen disordered-mixture double salt

**DOI:** 10.1107/S2056989015017703

**Published:** 2015-09-26

**Authors:** Hea-Chung Joo, Ki-Min Park, Uk Lee

**Affiliations:** a Department of Chemistry, Pukyong National University, 599-1 Daeyeon 3-dong, Nam-gu, Busan 608-737, Republic of Korea; b Research Institute of Natural Science, Gyeongsang National University, 501, Jinju-daero, Jinju, 660-701, Republic of Korea

**Keywords:** crystal structure, platinium containing polyoxomolybdate, double salt-type heteropolyoxometalate, hexa­molybdoplatinate(IV), multi­hydrogen bond

## Abstract

The title double salt containing two distinct, differently protonated hexa­molybdoplatinate(IV) polyanions, Na_6_[H_5.5_ *α*-PtMo_6_O_24_][H_4.5_ *α*-PtMo_6_O_24_]·29H_2_O, has been synthesized by a hydro­thermal reaction at *ca* pH 1.80. The positions of the H atoms in the polyanions were established from difference Fourier maps and confirmed by the inter­polyanion hydrogen bonds, bond-distance elongation, and bond-valence sum (BVS) calculations. The fractional numbers of H atoms in each polyanion are required for charge balance and in order to avoid unrealistically short H⋯H distances in the inter­polyanion hydrogen bonds. Considering the disorder, the refined formula of the title polyanion, {[H_5.5_ *α*-PtMo_6_O_24_]; polyanion (*A*) and [H_4.5_ *α*-PtMo_6_O_24_]; polyanion (*B*)}^6−^, can be rewritten as a set of real formula, *viz*. {[H_6_ *α*-PtMo_6_O_24_]; polyanion (*A*). [H_4_ *α*-PtMo_6_O_24_]; polyanion (*B*)}^6−^ and {[H_5_ *α*-PtMo_6_O_24_]; polyanion (*A*). [H_5_ *α*-PtMo_6_O_24_]; polyanion (*B*)}^6−^. The polyanion pairs both form dimers of the same formula, *viz*. {[H_10_ *α*-Pt2Mo_12_O_48_]}^6−^ connected by seven inter­polyanion O—H⋯O hydrogen bonds.

## Abstract 2

The title double salt containing two distinct, differently protonated hexa­molybdoplatinate(IV) polyanions. The polyanion pairs both form dimers of the same formula, *viz*. {[H_10_ *α*-Pt2Mo_12_O_48_]}^6−^ connected by seven inter­polyanion O—H⋯O hydrogen bonds.

## Chemical context   

The *α* (planar structure) – *β* (bent structure) – *α* geometrical isomerization, according to stepwise protonation in the [PtMo_6_O_24_]^8−^ polyoxometalate (POM) species, *viz*. ([H_3.5_ *α*-PtMo_6_O_24_]^4.5−^ (Lee & Sasaki, 1994 ▸; Lee, 1988 ▸) , [H_4_ *β*-PtMo_6_O_24_]^4−^ (Lee & Sasaki, 1994 ▸; Joo *et al.*, 1994 ▸) and [H_4.5_ *α*-PtMo_6_O_24_]^3.5−^ (Lee & Sasaki, 1994 ▸; Lee *et al.*, 2010 ▸; Joo *et al.*, 2015*a* ▸) is an unprecedented phenomenon in the Anderson-type heteropolyanion (Anderson, 1937 ▸) and as well as in the chemistry of POMs. In addition, differently proton­ated polyanion species have been reported, *viz*. [H_2_ *α*-PtMo_6_O_24_]^6−^ (Lee & Joo, 2000 ▸; Lee & Joo, 2004 ▸), and [H_6_ *α*-PtMo_6_O_24_]^2−^ (Lee & Joo, 2006*a* ▸; Lee & Joo, 2006*b* ▸; Lee & Joo, 2010 ▸). These polyanions form dimers by effective inter­polyanion hydrogen bonds. Recently, a hydrogen-bonded hexa­molybdoplatinate(IV) tetra­mer, [(*α*-PtMo_6_O_24_)_4_H_23_]^9−^, and the trimers, [(*α*-PtMo_6_O_24_)_3_H_16_]^8−^ and [(*α*-PtMo_6_O_24_)_3_H_14_]^10−^ were reported as tetra-*n*-butyl­ammonium, and tetra-*n*-butyl­ammonium/tri­ethyl­ammonium salts, respectively (Day *et al.*, 2009 ▸).

In our studies of Anderson-type heteropolyoxotungstates containing Pt^IV^, [H*_n_α*-Pt^IV^W_6_O_24_]^(8–*n*)–^ *n* = 0, 2, 2.5, 3, 3.5), we have found out that the gradual protonation is also a typical character of these compounds (Izarova *et al.*, 2012 ▸). Furthermore, we have reported the stepwise protonation species in the nona­vanadoplatinate(IV) series, *viz*. [H_*n*_PtV_9_O_28_]^(7−*n*)−^ (*n* = 2 and 3) (Lee *et al.*, 2008 ▸; Joo *et al.*, 2011 ▸; Joo & Lee, 2015 ▸; Joo *et al.*, 2015*b* ▸). As well as the Pt^IV^ a Keggin-type (Keggin, 1934 ▸) heteropolyoxometalate was formed, [*α*-SiPt^IV^_2_W_10_O_40_]^8−^ (Lee *et al.*, 2003 ▸).

The Pt^IV^ ion shows a very rich chemical behavior when it forms POMs with Mo, W and V systems. We assume that the diversity of the Pt^IV^-containing POMs is caused by the starting material of the heteroatom, [Pt^IV^(OH)_6_]^2−^, and the similarities in the oxidation states and the ionic radii of addenda atoms (Pt^4+^; 0.76, Mo^6+^; 0.73, W^6+^; 0.74 & V^5+^; 0.68 Å; Shannon, 1976 ▸) and the electron configuration of Pt^4+^ (5*d* ^6^) that preferentially forms the six-coordinated octa­hedra. In partic­ular, the selective protonation of the *μ*_3_-O atoms around Pt atom in the POMs is an important factor to the formation of POMs because the geometries of *M*—*μ*_3_-O (bond distance) and *M*—*μ*_3_-O—*M* (bond angle) (*M* = Mo, W and V) are changeable by the partial protonation of the *μ*_3_-O and *μ*_2_-O atoms.

## Structural commentary   

**Figure 1:**
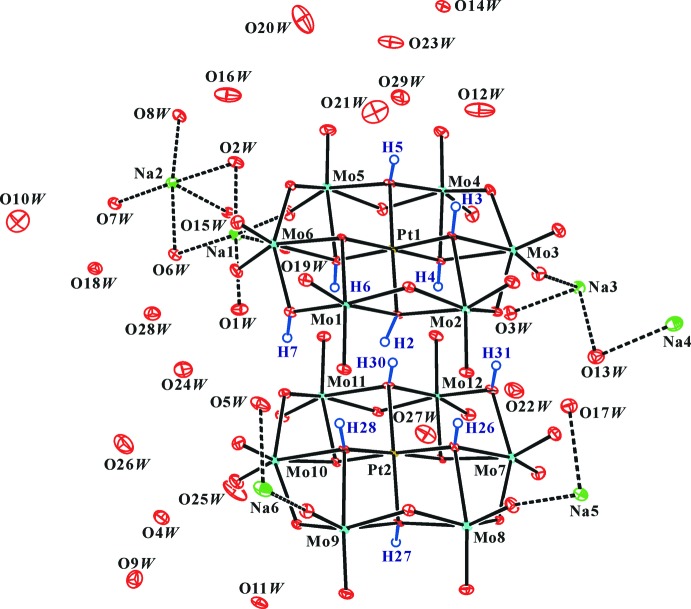
The mol­ecular entities in the crystal structure of the title compound. Displacement ellipsoids are drawn at the 50% probability level. The H atoms of the polyanion are presented as small spheres of arbitrary radius and the H atoms of water mol­ecules have been omitted for clarity. Bonds between coordinating O*W* molecules and Na^+^ are indicated by dashed lines.

The title compound contains two statistically different protonated hexa­molybdoplatinate(IV) polyanions, [H_5.5_ *α*-Pt^IV^Mo_5_O_24_]^2.5−^ (*A*), and [H_4.5_ *α*-Pt^IV^Mo_5_O_24_]^3.5−^ (*B*). Figs. 1 ▸ and 2 ▸ show the structures of the title compound and polyanions, respectively. The O atoms of the clusters were designated as O*T* (terminal Mo=O atom), O*B* (bridging *μ*_2_-O*B* atom; Mo—O—Mo), and O*C* (centered *μ*_3_-O atom; Mo_2_—O*C*—Pt).

**Figure 2:**
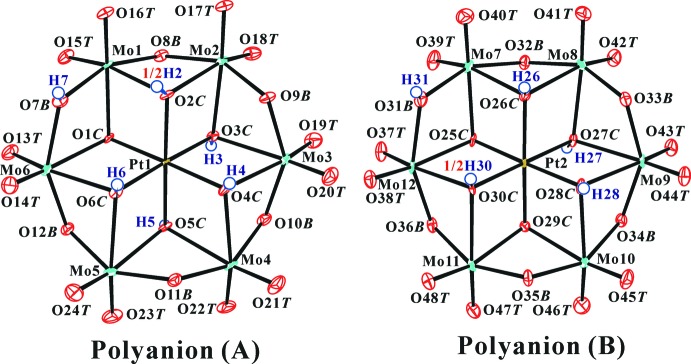
The polyanion structure in the title compound with the atomic numbering scheme and displacement ellipsoids at the 50% probability level for non-H atoms. H atoms are presented as small spheres of arbitrary radius.

**Figure 3:**
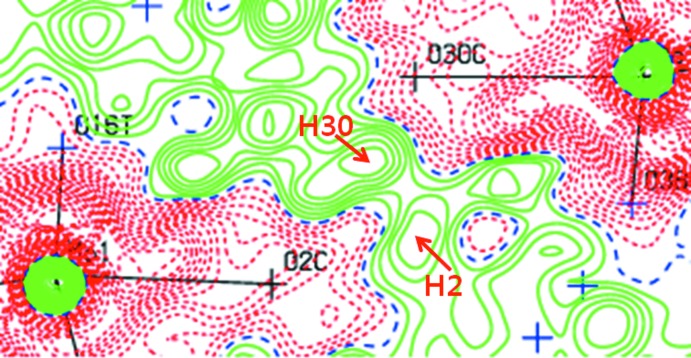
Difference-Fourier map around atoms H2 and H30. Calculated with atom H2 and H30 absent from the model.

**Figure 4:**
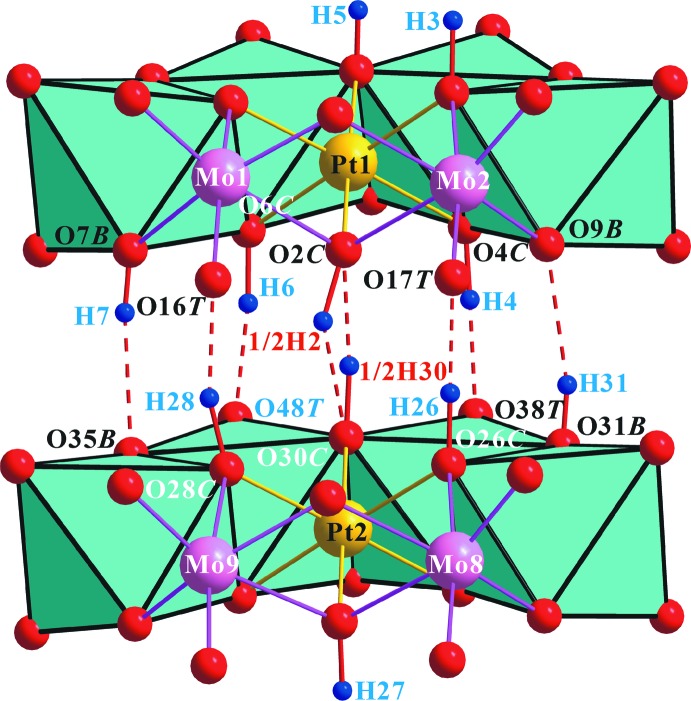
Polyhedral view of the heteropolyanion in the title compound, with O—H⋯O contacts of the inter­polyanion hydrogen bonds shown as red dashed lines. Disordered H atoms are included.

The H atoms of the protonated O atoms were found in difference Fourier maps and confirmed by bond-length elongation of Mo—O, and change of angles of Mo—O*B*—Mo and Mo—O*C*—Mo (Table 1 ▸), the inter­polyanion hydrogen bonds (Table 2 ▸ and Fig. 4), and the bond-valence sums (BVSs; Brown & Altermatt, 1985 ▸; Brese & O’Keeffe, 1991 ▸). The protonated O atoms in the hexa­molybdoplatinates(IV), polyanion (*A*) and (*B*), are five (Pt and Mo_2_)-bound *μ*_3_-O (O2*C*—O6*C*) and one Mo_2_-bound *μ*_2_-O (O7*B*) [for polyanion (*A*)], and four (Pt and Mo_2_)-bound *μ*_3_-O (O26*C*—O28*C* and O30*C*) and one Mo_2_-bound *μ*_2_-O (O31*B*) [for polyanion (*B*)] atoms. One (Pt and Mo_2_)-bound *μ*_3_-O atom in each polanion [O2*C* for polyanion (*A*) and O30*C* for polyanion (*B*)] is half-number protonated by disorder (Fig. 2 ▸). The residues of the two disordered H atoms, H2 and H30, were confirmed in the difference Fourier map (Fig. 3 ▸). This disorder is necessary for charge-balance of the polyanions and in order to avoid unreasonably short H⋯H distances in the inter­polyanion hydrogen bonds.

**Figure 5:**
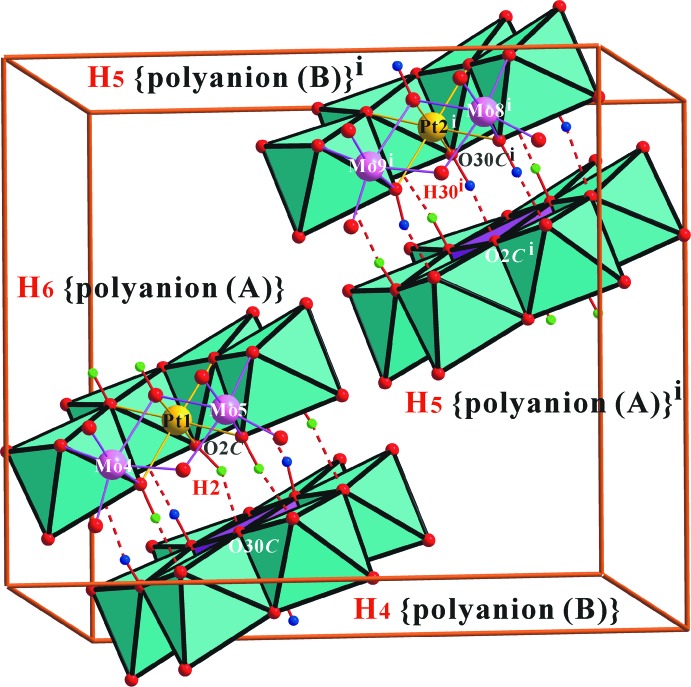
Polyhedral view of the unit-cell packing in the title compound, with O—H⋯O contacts of the inter­polyanion hydrogen bonds shown as red dashed lines. Disordered H atoms have been omitted. [Symmetry code: (i) −*x* + 1, −*y* + 1, −*z* + 1.]

Two discrete heteropolyanions, (*A*) and (*B*), form a dimer, {[H_10_ *α*-Pt_2_Mo_12_O_48_]^6−^, held together by two strong pairs of (Pt and Mo_2_)-bound *μ*_3_-O*C*—H⋯(Mo)-bound *μ*_1_-O*T*, normally a pair of (Mo2)-bound *μ*_2_-O*B*—H⋯(Mo_2_)-bound *μ*_2_-O*B*, and a single disordered strong (Pt and Mo_2_)-bound *μ*_3_-O*C*—H⋯(Pt and Mo2)-bound *μ*_3_-O*C* hydrogen bonds (Fig. 4 ▸ and Table 2 ▸). Considering the disorder, the statistically refined formula of the title polyanion, {[H_5.5_ *α*-PtMo_6_O_24_]·[H_4.5_ *α*-PtMo_6_O_24_]}^6−^, can be rewritten as mixture of dimers of {[H_6_ *α*-PtMo_6_O_24_]; polyanion (*A*)}·[H_4_ *α*-PtMo_6_O_24_]; polyanion (*B*)}^6−^ and {[H_5_ *α*-PtMo_6_O_24_]; polyanion (*A*)}·[H_5_ *α*-PtMo_6_O_24_]; polyanion (*B*)}^6−^ (Fig. 5 ▸). In other words, a set of polyanion (*A*), [H_5.5_ *α*-PtMo_6_O_24_]^2.5−^, and polyanion (*B*), [H_4.5_ *α*-PtMo_6_O_24_]^3.5−^, are the average disordered formulae of {[H_6_α-PtMo_6_O_24_]^2^·[H_4_α-PtMo_6_O_24_]^4−^} and {[H_5_α-PtMo_6_O_24_]^3−^·[H_5_α-PtMo_6_O_24_]^3−^} (Fig. 5 ▸).

The previously reported [*β*-H_4_PtMo_6_O_24_]^4−^ polyanion (Lee & Sasaki, 1994 ▸; Joo *et al.*, 1994 ▸) showed a bent structure (*C*_2*v*_) but the present polyanion shows a near planar structure. The protonated O atoms of [H_6_PtMo_6_O_24_]^2−^ in the present structure show the same protonation scheme as one previously reported (Lee & Joo, 2006*a* ▸,*b* ▸), *viz*. four *μ*_3_-O*C* and two *μ*_2_-O*B* atoms are protonated. However, the protonation scheme of the previously reported polyanion in [H_6_PtMo_6_O_24_]^2−^ (Lee & Joo, 2010 ▸) was different, consisting of five *μ*_3_-O*C* and one *μ*_2_-O*B* protonated O atoms. Five proton­ated polyanion species (*A*) and (*B*) were confirmed for the first time in the title compound. Four *μ*_3_-O and one *μ*_2_-O atoms are protonated in both polyanions, but the position of the unprotonated *μ*_3_-O atom differs (Fig. 2 ▸).

Confirmation of the protonated O atoms was strongly supported by the BVS analysis. The BVSs for protonated atoms O2*C*–O6*C* and O7*B* in polyanion (*A*) are 1.58, 1.45, 1.43, 1.36, 1.42 and 1.24, and O26*C*–O28*C*, O30*C* and O31*B* in the polyanion (*B*) are 1.41, 1.41, 1.39, 1.33 and 1.24 valence units (v.u.), respectively, if the valence of the O—H bond is not included. As the BVS value around the O atoms in the polyanion should be 2.0 v.u., the missing valences for each of the O atoms are 0.42 (for O2*C*), 0.55 (for O3*C*), 0.57 (for O4*C*), 0.64 (for O5*C*), 0.58 (for O6*C*) and 0.76 (for O7*B*) v.u. in polyanion (*A*), and 0.59 (for O26*C*), 0.59 (for O27*C*), 0.61 (for O28*C*), 0.67 (for O30*C*) and 0.76 (for O31*B*) in polyanion (*B*), respectively, corresponding to the valence of the O—H bonds. The BVSs around the other unprotonated atoms, O1*C* and O8*B*–O12*B* in the polyanion (A) and O25*C*, O29*C* and O32*B*–O36*B* in polyanion (*B*) are 1.82, 1.93, 1.84, 1.85, 1.90 and 1.90, and 1.82, 1.80, 1.94, 1.80, 1.81, 1.70 and 1.94 v.u., respectively, if the valence of the O*B* and the *C*⋯H—O*W* hydrogen bonds and (O*B* and *C*)⋯Na^+^ inter­actions are not included.

All Na^+^ cations are located on general positions of the space group *P* 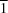. The calculated BVSs for the Na1–Na6 ions are 1.22, 1.19, 1.32, 1.10, 1.21 and 1.18 v.u., respectively (Na^+^⋯O distance 〈 2.50 Å; total v.u = 7.22). The Na^+^ ions are variously coordinated by O atoms as [Na1(O*T*)_2_(O*W*)_4_]^+^, [Na2(O*T*)(O*W*)_5_]^+^, [Na3(O*T*)_2_(O*W*)_4_]^+^, [Na4(O*T*)(O*W*)_4_]^+^, [Na5(O*T*)_2_(O*W*)_4_]^+^ and [Na6(O*T*)_2_(O*W*)_4_]^+^.

## Supra­molecular features   

The dimerized polyanions (*A*) + (*B*), {[H_10_ *α*-Pt_2_Mo_12_O_48_]^6−^, are connected three-dimensionally by O atoms of the polyanion coordinated to Na^+^ ions. Two discrete heteropolyanions, (*A*) and (*B*), form a dimer, {[H_10_ *α*-Pt_2_Mo_12_O_48_]^6−^, held together by two strong pairs of (Pt and Mo_2_)-bound *μ*_3_-O*C*—H⋯(Mo)-bound *μ*_1_-O*T*, normally a pair of (Mo_2_)-bound *μ*_2_-O*B*—H⋯(Mo_2_)-bound *μ*_2_-O*B*, and a single disordered strong (Pt and Mo_2_)-bound *μ*_3_O-*C*—H_0.5_⋯(Pt & Mo_2_)-bound *μ*_3_-O*C* hydrogen bond (Fig. 4 ▸ and Table 2 ▸). It is notable that the water mol­ecules O21*W*–O29*W*, do not show any inter­action with the metal atoms and are bonded to other O atoms only by O—H⋯O hydrogen bonds. The other H atoms of the polyanion (H3, H5 and H27) form hydrogen bonds with water mol­ecules (Table 2 ▸).

## Synthesis and crystallization   

Crystals of title compound were prepared by the reaction of Na_2_MoO_4_·2H_2_O and Na_2_Pt(OH)_6_ at *ca* pH 1.80 as described in a previous report (Lee & Sasaki, 1994 ▸).

## Refinement   

The crystal data, the data collection and the structure refinement details are summarized in Table 3 ▸. Atoms O5*C* and O30*C*, and O2*C* and O25*C* sets required an ISOR restraint in *SHELXL2014/7* (Sheldrick, 2015 ▸) with reduced deviation *s* = 0.004 and *st* = 0.008, and *s* = 0.002 and *st* = 0.004, respectively. All H atoms of polyanions were located in difference Fourier maps, and were refined with a distance restraint of O—H = 0.85 (3) Å using the command DFIX in *SHELXL2014/7*, and included in the refinement with *U*_iso_(H) = 1.5*U*_eq_(O). The occupancies of atoms H2 and H30 were reduced to 0.5 because of disorder. All H atoms of the water mol­ecules, except O12*W*–O15*W*, were located in difference Fourier maps, and were refined using a distance restraint of O—H = 0.85 (3) Å and an angle restraint of H*A*—H*B* = 1.40 (3) Å using the command DFIX in *SHELXL2014/7*, and included in the refinement with *U*_iso_(H) = 1.5*U*_eq_(O). An angle restraint of 1.35 (3) Å for O5*W*, O18*W* and O19*W*, and 1.30 (3) Å for O7*W* was applied. The H atoms of O12*W*–O13*W* were positioned geometrically and refined using a riding model (HFIX 137), with O*W*—H = 0.98 Å and *U*_iso_(H) = 1.5*U*_eq_(O). The H atoms of O14*W* were refined using a riding model (HFIX 23), with O*W*—H = 0.99 Å and *U*_iso_(H) = 1.5*U*_eq_(O). All invalid H atoms were removed in the final step of refinement. The highest peak in the difference map is 0.82 Å from Pt1 and the deepest hole is 0.98 Å from Pt2.

## Supplementary Material

Crystal structure: contains datablock(s) New_Global_Publ_Block, I. DOI: 10.1107/S2056989015017703/hb7461sup1.cif

Structure factors: contains datablock(s) I. DOI: 10.1107/S2056989015017703/hb7461Isup2.hkl

CCDC reference: 1426214

Additional supporting information:  crystallographic information; 3D view; checkCIF report

## Figures and Tables

**Table 1:** Selected geometric parameters (, )

Mo1O1*C*	2.114(3)	Mo1O7*B*	2.098(3)
Mo6O1*C*	2.198(3)	Mo6O7*B*	2.076(3)
Mo1O2*C*	2.216(3)	Mo1O8*B*	1.883(3)
Mo2O2*C*	2.246(3)	Mo2O8*B*	1.963(3)
Mo2O3*C*	2.245(3)	Mo2O9*B*	1.924(3)
Mo3O3*C*	2.336(3)	Mo3O9*B*	1.953(3)
Mo3O4*C*	2.267(3)	Mo3O10*B*	1.927(3)
Mo4O4*C*	2.283(3)	Mo4O10*B*	1.947(3)
Mo4O5*C*	2.312(3)	Mo4O11*B*	1.916(3)
Mo5O5*C*	2.280(3)	Mo5O11*B*	1.935(3)
Mo5O6*C*	2.358(3)	Mo5O12*B*	1.947(3)
Mo6O6*C*	2.287(3)	Mo6O12*B*	1.906(3)
Mo7O25*C*	2.186(3)	Mo7O31*B*	2.072(3)
Mo12O25*C*	2.084(3)	Mo12O31*B*	2.090(3)
Mo7O26*C*	2.297(3)	Mo7O32*B*	1.899(3)
Mo8O26*C*	2.305(3)	Mo8O32*B*	1.935(3)
Mo8O27*C*	2.272(3)	Mo8O33*B*	1.959(3)
Mo9O27*C*	2.302(3)	Mo9O33*B*	1.932(3)
Mo9O28*C*	2.307(3)	Mo9O34*B*	1.925(3)
Mo10O28*C*	2.302(3)	Mo10O34*B*	1.961(3)
Mo10O29*C*	2.196(3)	Mo10O35*B*	1.988(3)
Mo11O29*C*	2.122(3)	Mo11O35*B*	1.947(3)
Mo11O30*C*	2.359(3)	Mo11O36*B*	1.970(3)
Mo12O30*C*	2.340(3)	Mo12O36*B*	1.870(3)
			
Mo1O1*C*Mo6	104.42(13)	Mo12O25*C*Mo7	104.38(13)
Mo1O2*C*Mo2	93.27(12)	Mo7O26*C*Mo8	92.23(11)
Mo2O3*C*Mo3	92.61(11)	Mo8O27*C*Mo9	93.67(11)
Mo3O4*C*Mo4	94.36(12)	Mo10O28*C*Mo9	93.85(11)
Mo5O5*C*Mo4	93.05(11)	Mo11O29*C*Mo10	96.01(12)
Mo6O6*C*Mo5	91.98(11)	Mo12O30*C*Mo11	90.63(11)
Mo6O7*B*Mo1	109.51(15)	Mo7O31*B*Mo12	108.35(15)
Mo1O8*B*Mo2	115.01(15)	Mo7O32*B*Mo8	119.84(16)
Mo2O9*B*Mo3	117.38(16)	Mo9O33*B*Mo8	118.01(15)
Mo3O10*B*Mo4	118.97(16)	Mo9O34*B*Mo10	120.01(16)
Mo4O11*B*Mo5	119.86(16)	Mo11O35*B*Mo10	109.31(15)
Mo6O12*B*Mo5	120.24(16)	Mo12O36*B*Mo11	120.93(15)

**Table 2:** Hydrogen-bond geometry (, )

*D*H*A*	*D*H	H*A*	*D* *A*	*D*H*A*
O2*C*H2O30*C*	0.86(3)	1.84(6)	2.595(5)	145(9)
O3*C*H3O14*W* ^i^	0.86(3)	1.74(3)	2.586(5)	164(4)
O4*C*H4O38*T*	0.85(3)	1.72(3)	2.576(4)	178(5)
O5*C*H5O29*W*	0.86(3)	1.79(3)	2.595(5)	156(5)
O6*C*H6O48*T*	0.86(3)	1.72(3)	2.569(4)	171(5)
O7*B*H7O35*B*	0.84(3)	1.94(3)	2.785(5)	175(5)
O26*C*H26O17*T*	0.82(3)	1.73(3)	2.556(4)	178(5)
O27*C*H27O15*W* ^ii^	0.87(3)	1.70(3)	2.548(5)	164(5)
O28*C*H28O16*T*	0.86(3)	1.73(3)	2.575(4)	166(5)
O30*C*H30O2*C*	0.84(3)	1.76(3)	2.595(5)	172(9)
O31*B*H31O9*B*	0.82(3)	1.95(3)	2.763(4)	171(5)
O1*W*H1*A*O36*B* ^ii^	0.87(3)	1.96(3)	2.830(5)	173(5)
O1*W*H1*B*O48*T*	0.84(3)	2.22(3)	3.023(5)	161(5)
O2*W*H2*A*O16*W*	0.86(3)	1.87(3)	2.731(6)	175(5)
O2*W*H2*B*O43*T* ^iii^	0.87(3)	2.17(3)	3.031(5)	169(5)
O3*W*H3*A*O22*W*	0.87(3)	2.10(3)	2.969(6)	175(5)
O3*W*H3*B*O38*T*	0.86(3)	2.13(3)	2.980(5)	176(5)
O4*W*H4*A*O26*W*	0.88(3)	1.98(3)	2.857(6)	175(5)
O4*W*H4*B*O25*W*	0.82(3)	1.99(3)	2.806(6)	176(6)
O5*W*H5*A*O16*T*	0.85(3)	2.08(3)	2.930(5)	178(6)
O6*W*H6*A*O25*C* ^ii^	0.86(3)	2.04(3)	2.880(5)	167(5)
O6*W*H6*B*O18*W*	0.87(3)	1.97(3)	2.831(6)	173(5)
O7*W*H7*A*O17*W* ^iv^	0.82(3)	1.98(3)	2.804(5)	175(6)
O7*W*H7*B*O32*B* ^ii^	0.82(3)	2.02(3)	2.842(5)	174(5)
O8*W*H8*A*O34*B* ^iii^	0.87(3)	2.21(3)	3.064(5)	168(5)
O8*W*H8*B*O9*W* ^iii^	0.87(3)	1.89(3)	2.750(6)	169(5)
O9*W*H9*A*O19*T* ^v^	0.83(3)	2.22(3)	3.046(5)	178(5)
O9*W*H9*B*O7*W* ^vi^	0.86(3)	1.91(3)	2.720(5)	156(6)
O10*W*H10*A*O25*W* ^vii^	0.84(3)	2.23(4)	2.924(7)	140(5)
O10*W*H10*B*O21*W* ^viii^	0.86(3)	2.02(3)	2.874(7)	174(7)
O11*W*H11*A*O34*B*	0.85(3)	1.93(3)	2.723(5)	155(5)
O11*W*H11*B*O43*T* ^ix^	0.85(3)	2.08(3)	2.867(5)	154(5)
O12*W*H12*A*O22*T*	0.98	2.25	2.904(5)	123
O12*W*H12*A*O21*W*	0.98	2.31	3.141(8)	142
O13*W*H13*A*O12*W* ^x^	0.99	1.80	2.766(6)	164
O13*W*H13*B*O31*B*	0.99	2.53	3.396(5)	146
O14*W*H14*A*O27*W* ^iii^	0.98	1.76	2.737(6)	177
O14*W*H14*B*O23*W*	0.98	1.96	2.796(6)	142
O15*W*H15*A*O19*W*	0.83(3)	1.97(3)	2.738(5)	154(5)
O16*W*H16*A*O20*W*	0.89(3)	2.45(6)	3.156(7)	137(7)
O16*W*H16*B*O24*W* ^viii^	0.85(3)	2.17(6)	2.842(6)	136(6)
O17*W*H17*A*O8*B* ^xi^	0.81(3)	1.98(3)	2.790(5)	173(5)
O17*W*H17*B*O17*T*	0.84(3)	2.22(3)	3.027(5)	160(5)
O18*W*H18*A*O28*W*	0.83(3)	2.18(4)	2.907(5)	146(5)
O18*W*H18*B*O1*C* ^viii^	0.81(3)	1.99(3)	2.798(5)	176(5)
O19*W*H19*A*O29*C* ^ii^	0.85(3)	2.01(3)	2.842(5)	164(5)
O19*W*H19*B*O10*W* ^viii^	0.80(3)	2.14(3)	2.920(6)	164(6)
O20*W*H20*B*O23*W*	0.85(3)	2.46(7)	3.121(8)	135(8)
O21*W*H21*A*O23*T*	0.89(3)	2.24(4)	3.064(6)	155(7)
O21*W*H21*B*O33*B* ^iii^	0.86(3)	2.17(3)	2.972(5)	155(6)
O22*W*H22*A*O28*W* ^ii^	0.88(3)	2.28(5)	3.007(6)	140(5)
O22*W*H22*B*O26*W* ^ii^	0.85(3)	1.96(3)	2.805(7)	169(6)
O23*W*H23*A*O22*T*	0.87(3)	2.30(5)	2.970(6)	134(6)
O23*W*H23*B*O10*B* ^i^	0.85(3)	1.95(3)	2.775(5)	162(7)
O24*W*H24*A*O28*W*	0.84(3)	2.02(3)	2.854(6)	173(6)
O24*W*H24*B*O35*B*	0.89(3)	2.05(3)	2.911(5)	163(5)
O25*W*H25*A*O38*T* ^ii^	0.83(3)	2.52(6)	3.119(6)	130(7)
O25*W*H25*B*O47*T*	0.86(3)	2.01(3)	2.834(5)	161(7)
O26*W*H26*A*O24*W*	0.87(3)	1.93(5)	2.723(6)	150(7)
O26*W*H26*B*O19*T* ^v^	0.87(3)	2.23(4)	2.920(5)	135(5)
O27*W*H27*A*O18*T* ^xi^	0.86(3)	2.33(5)	2.945(5)	129(5)
O27*W*H27*B*O33*B*	0.87(3)	2.10(4)	2.846(5)	144(5)
O28*W*H28*A*O12*B* ^viii^	0.86(3)	1.93(3)	2.775(5)	168(6)
O28*W*H28*B*O1*W*	0.86(3)	1.96(3)	2.817(6)	171(6)
O29*W*H29*A*O22*T* ^i^	0.86(3)	2.26(5)	2.895(5)	131(5)
O29*W*H29*B*O22*W* ^vii^	0.84(3)	2.03(3)	2.844(6)	165(7)

Symmetry codes: (i) 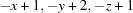; (ii) 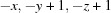; (iii) 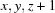; (iv) 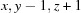; (v) 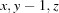; (vi) 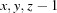; (vii) 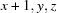; (viii) 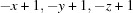; (ix) 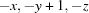; (x) 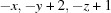; (xi) 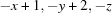.

**Table 3:** Experimental details

Crystal data	
Chemical formula	Na_6_[H_5.5_ **-PtMo_6_O_24_]
*M*_r_	2979.85
Crystal system, space group	Triclinic, *P* 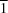
Temperature (K)	173
*a*, *b*, *c* ()	14.0384(6), 15.7969(6), 16.7235(6)
, , ()	72.825(2), 75.522(2), 89.168(2)
*V* (^3^)	3423.7(2)
*Z*	2
Radiation type	Mo *K*
(mm^1^)	6.36
Crystal size (mm)	0.67 0.44 0.22

Data collection	
Diffractometer	Bruker SMART APEXII CCD
Absorption correction	Multi-scan (*SADABS*; Bruker, 2009 ▸)
*T*_min_, *T*_max_	0.234, 0.746
No. of measured, independent and observed [*I* > 2(*I*)] reflections	58415, 14940, 12688
*R*_int_	0.057
(sin /)_max_ (^1^)	0.639

Refinement	
*R*[*F* ^2^ > 2(*F* ^2^)], *wR*(*F* ^2^), *S*	0.036, 0.091, 1.06
No. of reflections	14940
No. of parameters	1064
No. of restraints	114
H-atom treatment	Only H-atom coordinates refined
_max_, _min_ (e ^3^)	1.73, 2.25

Computer programs: *APEX2* and *SAINT* (Bruker, 2009 ▸), *SHELXS2014/7* (Sheldrick, 2008 ▸), *SHELXL2014/7* (Sheldrick, 2015 ▸), *ORTEP-3 for Windows* (Farrugia, 2012 ▸), *PLATON* (Spek, 2009 ▸) and *DIAMOND* (Brandenburg, 1998 ▸).

## supplementary crystallographic information

### Crystal data

**Table:**

H_6_Mo_6_O_24_Pt·H_4_Mo_6_O_24_Pt·29(H_2_O)·6(Na)	*Z* = 2
*M**_r_* = 2979.85	*F*(000) = 2820
Triclinic, *P*1	*D*_x_ = 2.891 Mg m^−^^3^
*a* = 14.0384 (6) Å	Mo *K*α radiation, λ = 0.71073 Å
*b* = 15.7969 (6) Å	Cell parameters from 9847 reflections
*c* = 16.7235 (6) Å	θ = 2.2–28.3°
α = 72.825 (2)°	µ = 6.36 mm^−^^1^
β = 75.522 (2)°	*T* = 173 K
γ = 89.168 (2)°	Block, yellow
*V* = 3423.7 (2) Å^3^	0.67 × 0.44 × 0.22 mm

### Data collection

**Table:**

Bruker SMART APEXII CCD diffractometer	14940 independent reflections
Radiation source: Rotating Anode	12688 reflections with *I* > 2σ(*I*)
Graphite multilayer monochromator	*R*_int_ = 0.057
Detector resolution: 10.0 pixels mm^-1^	θ_max_ = 27.0°, θ_min_ = 1.4°
φ and ω scans	*h* = −16→17
Absorption correction: multi-scan (*SADABS*; Bruker, 2009)	*k* = −20→20
*T*_min_ = 0.234, *T*_max_ = 0.746	*l* = −21→21
58415 measured reflections	

### Refinement

**Table:**

Refinement on *F*^2^	Secondary atom site location: difference Fourier map
Least-squares matrix: full	Hydrogen site location: difference Fourier map
*R*[*F*^2^ > 2σ(*F*^2^)] = 0.036	Only H-atom coordinates refined
*wR*(*F*^2^) = 0.091	*w* = 1/[σ^2^(*F*_o_^2^) + (0.0223*P*)^2^ + 5.6226*P*] where *P* = (*F*_o_^2^ + 2*F*_c_^2^)/3
*S* = 1.06	(Δ/σ)_max_ = 0.002
14940 reflections	Δρ_max_ = 1.73 e Å^−^^3^
1064 parameters	Δρ_min_ = −2.25 e Å^−^^3^
114 restraints	Extinction correction: *SHELXL2014*/7 (Sheldrick 2014, Fc^*^=kFc[1+0.001xFc^2^λ^3^/sin(2θ)]^-1/4^
Primary atom site location: structure-invariant direct methods	Extinction coefficient: 0.00097 (2)

### Special details

**Table:**

Geometry. All esds (except the esd in the dihedral angle between two l.s. planes) are estimated using the full covariance matrix. The cell esds are taken into account individually in the estimation of esds in distances, angles and torsion angles; correlations between esds in cell parameters are only used when they are defined by crystal symmetry. An approximate (isotropic) treatment of cell esds is used for estimating esds involving l.s. planes.

### Fractional atomic coordinates and isotropic or equivalent isotropic displacement parameters (Å^2^)

**Table:**

	*x*	*y*	*z*	*U*_iso_*/*U*_eq_	Occ. (<1)
Pt1	0.37861 (2)	0.80827 (2)	0.33415 (2)	0.00743 (6)	
Pt2	0.13101 (2)	0.69756 (2)	0.15934 (2)	0.00734 (6)	
Mo1	0.48088 (3)	0.73755 (3)	0.17456 (2)	0.01032 (9)	
Mo2	0.38120 (3)	0.92698 (3)	0.13444 (2)	0.01043 (9)	
Mo3	0.27924 (3)	1.00756 (3)	0.29553 (3)	0.01189 (9)	
Mo4	0.27941 (3)	0.89367 (3)	0.49832 (2)	0.01226 (9)	
Mo5	0.37444 (3)	0.69579 (3)	0.54288 (2)	0.01302 (9)	
Mo6	0.47496 (3)	0.61324 (3)	0.38076 (3)	0.01233 (9)	
Mo7	0.02597 (3)	0.89077 (3)	0.11926 (3)	0.01261 (9)	
Mo8	0.12559 (3)	0.81350 (3)	−0.04502 (2)	0.01205 (9)	
Mo9	0.22090 (3)	0.61525 (3)	−0.00919 (2)	0.01097 (9)	
Mo10	0.21502 (3)	0.49586 (3)	0.19550 (2)	0.01178 (9)	
Mo11	0.12327 (3)	0.56971 (3)	0.35600 (2)	0.01106 (9)	
Mo12	0.02151 (3)	0.76546 (3)	0.32235 (2)	0.01089 (9)	
Na1	0.23742 (14)	0.46059 (13)	0.69299 (12)	0.0204 (4)	
Na2	0.23737 (13)	0.33338 (13)	0.89570 (12)	0.0215 (4)	
Na3	0.00711 (13)	1.06967 (13)	0.39992 (12)	0.0207 (4)	
Na4	0.02805 (15)	1.23682 (14)	0.20474 (13)	0.0312 (5)	
Na5	0.25851 (14)	1.04851 (13)	−0.20594 (12)	0.0197 (4)	
Na6	0.50542 (14)	0.56953 (15)	−0.10815 (14)	0.0302 (5)	
O1C	0.4984 (2)	0.7498 (2)	0.29268 (19)	0.0093 (6)	
O2C	0.3429 (2)	0.7918 (2)	0.2319 (2)	0.0114 (7)	
H2	0.302 (6)	0.750 (4)	0.236 (6)	0.017*	0.5
O3C	0.4185 (2)	0.9314 (2)	0.25614 (19)	0.0098 (7)	
H3	0.470 (2)	0.958 (3)	0.260 (3)	0.015*	
O4C	0.2544 (2)	0.8638 (2)	0.3794 (2)	0.0116 (7)	
H4	0.199 (2)	0.840 (3)	0.382 (3)	0.017*	
O5C	0.4156 (2)	0.8287 (2)	0.43710 (19)	0.0117 (7)	
H5	0.471 (2)	0.858 (3)	0.423 (3)	0.018*	
O6C	0.3372 (2)	0.6876 (2)	0.4153 (2)	0.0115 (7)	
H6	0.283 (2)	0.664 (3)	0.415 (3)	0.017*	
O7B	0.4252 (2)	0.6188 (2)	0.2725 (2)	0.0165 (7)	
H7	0.372 (3)	0.593 (3)	0.275 (3)	0.025*	
O8B	0.5009 (2)	0.8611 (2)	0.12156 (19)	0.0137 (7)	
O9B	0.2635 (2)	0.9556 (2)	0.20675 (19)	0.0146 (7)	
O10B	0.3285 (2)	0.9949 (2)	0.3959 (2)	0.0157 (7)	
O11B	0.2613 (2)	0.7673 (2)	0.54830 (19)	0.0155 (7)	
O12B	0.4894 (2)	0.6608 (2)	0.4697 (2)	0.0144 (7)	
O13T	0.5925 (2)	0.5883 (2)	0.3436 (2)	0.0215 (8)	
O14T	0.4079 (3)	0.5158 (2)	0.4360 (2)	0.0232 (8)	
O15T	0.5986 (2)	0.7061 (2)	0.1477 (2)	0.0187 (8)	
O16T	0.4206 (2)	0.7054 (2)	0.1085 (2)	0.0165 (7)	
O17T	0.3308 (2)	0.9010 (2)	0.0596 (2)	0.0163 (7)	
O18T	0.4392 (2)	1.0292 (2)	0.0834 (2)	0.0199 (8)	
O19T	0.3365 (3)	1.1085 (2)	0.2315 (2)	0.0230 (8)	
O20T	0.1590 (2)	1.0260 (2)	0.3325 (2)	0.0228 (8)	
O21T	0.1608 (2)	0.9179 (2)	0.5323 (2)	0.0250 (9)	
O22T	0.3430 (3)	0.9230 (2)	0.5618 (2)	0.0225 (8)	
O23T	0.4295 (3)	0.7258 (3)	0.6111 (2)	0.0262 (9)	
O24T	0.3127 (3)	0.5959 (2)	0.5988 (2)	0.0247 (8)	
O25C	0.0097 (2)	0.7540 (2)	0.20418 (19)	0.0101 (7)	
O26C	0.1662 (2)	0.8208 (2)	0.0782 (2)	0.0107 (7)	
H26	0.220 (2)	0.846 (3)	0.073 (3)	0.016*	
O27C	0.0895 (2)	0.6796 (2)	0.05850 (19)	0.0106 (7)	
H27	0.030 (2)	0.655 (3)	0.075 (3)	0.016*	
O28C	0.2520 (2)	0.6413 (2)	0.1108 (2)	0.0103 (7)	
H28	0.311 (2)	0.654 (3)	0.111 (3)	0.015*	
O29C	0.0931 (2)	0.5750 (2)	0.23650 (19)	0.0108 (7)	
O30C	0.1708 (2)	0.7142 (2)	0.26046 (19)	0.0119 (7)	
H30	0.224 (4)	0.744 (5)	0.250 (6)	0.018*	0.5
O31B	0.0771 (2)	0.8849 (2)	0.2266 (2)	0.0165 (7)	
H31	0.129 (3)	0.910 (3)	0.223 (3)	0.025*	
O32B	0.0110 (2)	0.8455 (2)	0.0295 (2)	0.0150 (7)	
O33B	0.2413 (2)	0.7429 (2)	−0.05672 (19)	0.0144 (7)	
O34B	0.1690 (2)	0.5144 (2)	0.09047 (19)	0.0141 (7)	
O35B	0.2443 (2)	0.5361 (2)	0.28926 (19)	0.0147 (7)	
O36B	0.0079 (2)	0.6417 (2)	0.36859 (19)	0.0132 (7)	
O37T	−0.0955 (2)	0.7942 (2)	0.3539 (2)	0.0219 (8)	
O38T	0.0849 (2)	0.7958 (2)	0.3885 (2)	0.0180 (8)	
O39T	−0.0918 (3)	0.9120 (3)	0.1604 (2)	0.0251 (9)	
O40T	0.0901 (3)	0.9901 (2)	0.0659 (2)	0.0265 (9)	
O41T	0.1871 (3)	0.9132 (2)	−0.1021 (2)	0.0230 (8)	
O42T	0.0681 (3)	0.7831 (2)	−0.1111 (2)	0.0219 (8)	
O43T	0.1597 (2)	0.5900 (2)	−0.0772 (2)	0.0198 (8)	
O44T	0.3380 (2)	0.5884 (2)	−0.0418 (2)	0.0210 (8)	
O45T	0.3332 (3)	0.4717 (2)	0.1580 (2)	0.0233 (8)	
O46T	0.1507 (3)	0.3978 (2)	0.2531 (2)	0.0264 (8)	
O47T	0.0646 (2)	0.4688 (2)	0.4142 (2)	0.0200 (8)	
O48T	0.1785 (2)	0.6017 (2)	0.4253 (2)	0.0169 (7)	
O1W	0.1853 (3)	0.4397 (3)	0.5748 (2)	0.0277 (9)	
H1A	0.124 (2)	0.419 (3)	0.591 (3)	0.042*	
H1B	0.195 (4)	0.479 (3)	0.527 (2)	0.042*	
O2W	0.2834 (3)	0.4879 (3)	0.8111 (2)	0.0258 (9)	
H2A	0.344 (2)	0.506 (3)	0.800 (3)	0.039*	
H2B	0.251 (3)	0.524 (3)	0.837 (3)	0.039*	
O3W	−0.0402 (3)	0.9185 (2)	0.4682 (2)	0.0248 (8)	
H3A	−0.1018 (19)	0.904 (4)	0.473 (3)	0.037*	
H3B	−0.003 (3)	0.883 (3)	0.447 (4)	0.037*	
O4W	0.0692 (3)	0.2145 (3)	0.3358 (3)	0.0327 (9)	
H4A	0.131 (2)	0.221 (4)	0.336 (4)	0.049*	
H4B	0.035 (3)	0.245 (4)	0.362 (4)	0.049*	
O5W	0.5335 (3)	0.5855 (3)	0.0218 (3)	0.0339 (10)	
H5A	0.500 (3)	0.621 (3)	0.046 (4)	0.051*	
H5B	0.591 (2)	0.601 (4)	0.007 (4)	0.051*	
O6W	0.1875 (3)	0.3127 (2)	0.7759 (2)	0.0207 (8)	
H6A	0.1274 (19)	0.292 (3)	0.790 (3)	0.031*	
H6B	0.225 (3)	0.273 (3)	0.760 (3)	0.031*	
O7W	0.1841 (3)	0.1846 (3)	0.9808 (3)	0.0267 (9)	
H7A	0.221 (3)	0.151 (3)	0.959 (4)	0.040*	
H7B	0.126 (2)	0.175 (4)	0.982 (4)	0.040*	
O8W	0.2657 (3)	0.3672 (2)	1.0195 (2)	0.0254 (8)	
H8A	0.247 (4)	0.411 (2)	1.040 (3)	0.038*	
H8B	0.239 (4)	0.320 (2)	1.061 (3)	0.038*	
O9W	0.1896 (3)	0.2051 (3)	0.1354 (3)	0.0366 (10)	
H9A	0.229 (4)	0.180 (4)	0.162 (3)	0.055*	
H9B	0.184 (4)	0.183 (4)	0.096 (3)	0.055*	
O10W	0.8819 (3)	0.2975 (3)	0.2745 (3)	0.0474 (11)	
H10A	0.875 (4)	0.287 (5)	0.328 (2)	0.071*	
H10B	0.832 (4)	0.276 (5)	0.263 (4)	0.071*	
O11W	0.0385 (3)	0.3872 (2)	0.0928 (2)	0.0261 (8)	
H11A	0.073 (3)	0.420 (3)	0.109 (3)	0.039*	
H11B	−0.022 (2)	0.398 (4)	0.103 (4)	0.039*	
O12W	0.2141 (3)	0.9781 (4)	0.7002 (3)	0.0618 (16)	
H12A	0.2596	0.9318	0.6923	0.093*	
H12B	0.2181	1.0222	0.6440	0.093*	
O13W	−0.0165 (3)	1.0739 (3)	0.2621 (2)	0.0315 (9)	
H13A	−0.0853	1.0581	0.2644	0.047*	
H13B	0.0300	1.0386	0.2321	0.047*	
O14W	0.4187 (2)	0.9806 (2)	0.7644 (2)	0.0233 (8)	
H14A	0.4196	0.9282	0.8133	0.035*	
H14B	0.4274	0.9631	0.7116	0.035*	
O15W	0.0789 (3)	0.3932 (2)	0.9223 (2)	0.0219 (8)	
H15A	0.073 (4)	0.439 (3)	0.884 (2)	0.033*	
H15B	0.075 (4)	0.399 (3)	0.9722 (19)	0.033*	
O16W	0.4775 (3)	0.5453 (4)	0.7651 (3)	0.0575 (15)	
H16A	0.461 (5)	0.600 (3)	0.762 (5)	0.086*	
H16B	0.518 (4)	0.550 (4)	0.717 (3)	0.086*	
O17W	0.3075 (3)	1.0648 (3)	−0.0822 (2)	0.0257 (9)	
H17A	0.362 (3)	1.089 (3)	−0.098 (3)	0.039*	
H17B	0.300 (4)	1.015 (2)	−0.043 (3)	0.039*	
O18W	0.3126 (3)	0.1933 (3)	0.7104 (2)	0.0250 (9)	
H18A	0.308 (4)	0.205 (4)	0.660 (2)	0.038*	
H18B	0.367 (3)	0.208 (4)	0.712 (3)	0.038*	
O19W	0.0830 (3)	0.5111 (3)	0.7640 (3)	0.0279 (9)	
H19A	0.032 (3)	0.493 (3)	0.754 (4)	0.042*	
H19B	0.093 (4)	0.5638 (18)	0.744 (4)	0.042*	
O20W	0.5152 (5)	0.7241 (3)	0.8036 (4)	0.0765 (17)	
H20A	0.540 (7)	0.731 (6)	0.845 (5)	0.115*	
H20B	0.483 (6)	0.769 (4)	0.785 (5)	0.115*	
O21W	0.2843 (4)	0.7843 (4)	0.7520 (3)	0.0685 (16)	
H21A	0.340 (4)	0.771 (5)	0.720 (4)	0.103*	
H21B	0.290 (5)	0.766 (6)	0.804 (2)	0.103*	
O22W	−0.2460 (4)	0.8598 (3)	0.4844 (3)	0.0504 (12)	
H22A	−0.246 (5)	0.824 (4)	0.452 (3)	0.076*	
H22B	−0.257 (6)	0.827 (4)	0.5371 (19)	0.076*	
O23W	0.5070 (3)	0.8893 (3)	0.6478 (3)	0.0476 (12)	
H23A	0.487 (4)	0.889 (5)	0.603 (3)	0.071*	
H23B	0.564 (3)	0.917 (4)	0.628 (4)	0.071*	
O24W	0.3421 (3)	0.3767 (3)	0.3558 (3)	0.0424 (11)	
H24A	0.339 (5)	0.358 (4)	0.409 (2)	0.064*	
H24B	0.305 (4)	0.423 (3)	0.347 (4)	0.064*	
O25W	−0.0526 (4)	0.3217 (3)	0.4196 (3)	0.0541 (14)	
H25A	−0.097 (4)	0.302 (4)	0.465 (3)	0.081*	
H25B	−0.029 (5)	0.373 (3)	0.416 (4)	0.081*	
O26W	0.2713 (3)	0.2255 (3)	0.3382 (3)	0.0518 (12)	
H26A	0.277 (5)	0.282 (2)	0.335 (4)	0.078*	
H26B	0.309 (5)	0.219 (4)	0.291 (3)	0.078*	
O27W	0.4268 (3)	0.8374 (3)	−0.0976 (3)	0.0378 (10)	
H27A	0.465 (4)	0.845 (4)	−0.067 (3)	0.057*	
H27B	0.379 (3)	0.799 (3)	−0.065 (3)	0.057*	
O28W	0.3135 (3)	0.3091 (3)	0.5390 (3)	0.0300 (9)	
H28A	0.373 (2)	0.326 (4)	0.533 (4)	0.045*	
H28B	0.277 (3)	0.348 (3)	0.555 (4)	0.045*	
O29W	0.5730 (3)	0.9091 (3)	0.4418 (3)	0.0440 (12)	
H29A	0.573 (5)	0.966 (2)	0.420 (4)	0.066*	
H29B	0.621 (4)	0.896 (4)	0.463 (4)	0.066*	

### Atomic displacement parameters (Å^2^)

**Table:**

	*U*^11^	*U*^22^	*U*^33^	*U*^12^	*U*^13^	*U*^23^
Pt1	0.00954 (10)	0.00867 (10)	0.00598 (9)	−0.00002 (7)	−0.00068 (6)	−0.00612 (7)
Pt2	0.00933 (10)	0.00833 (10)	0.00628 (9)	0.00006 (7)	−0.00122 (6)	−0.00569 (7)
Mo1	0.0110 (2)	0.0139 (2)	0.01004 (19)	0.00019 (16)	−0.00179 (15)	−0.01018 (16)
Mo2	0.0118 (2)	0.0125 (2)	0.00893 (19)	−0.00137 (16)	−0.00222 (14)	−0.00634 (16)
Mo3	0.0139 (2)	0.0111 (2)	0.0126 (2)	0.00246 (16)	−0.00246 (15)	−0.00733 (16)
Mo4	0.0147 (2)	0.0147 (2)	0.00968 (19)	0.00173 (16)	−0.00023 (15)	−0.00955 (17)
Mo5	0.0176 (2)	0.0135 (2)	0.00813 (19)	0.00016 (17)	−0.00184 (15)	−0.00471 (16)
Mo6	0.0149 (2)	0.0109 (2)	0.0134 (2)	0.00249 (16)	−0.00354 (15)	−0.00718 (17)
Mo7	0.0148 (2)	0.0111 (2)	0.0145 (2)	0.00282 (16)	−0.00414 (16)	−0.00732 (17)
Mo8	0.0162 (2)	0.0121 (2)	0.00841 (19)	−0.00019 (16)	−0.00287 (15)	−0.00410 (16)
Mo9	0.0123 (2)	0.0136 (2)	0.00969 (19)	0.00170 (16)	−0.00127 (15)	−0.00888 (16)
Mo10	0.0152 (2)	0.0105 (2)	0.0114 (2)	0.00199 (16)	−0.00271 (15)	−0.00665 (16)
Mo11	0.0133 (2)	0.0140 (2)	0.00731 (19)	−0.00197 (16)	−0.00172 (14)	−0.00598 (16)
Mo12	0.0122 (2)	0.0140 (2)	0.01044 (19)	0.00028 (16)	−0.00208 (15)	−0.01016 (17)
Na1	0.0246 (11)	0.0195 (10)	0.0167 (10)	−0.0017 (8)	−0.0031 (8)	−0.0068 (8)
Na2	0.0182 (10)	0.0275 (11)	0.0215 (10)	0.0043 (8)	−0.0049 (8)	−0.0117 (9)
Na3	0.0208 (11)	0.0216 (11)	0.0191 (10)	0.0018 (9)	−0.0013 (8)	−0.0083 (9)
Na4	0.0332 (12)	0.0398 (13)	0.0217 (11)	0.0021 (10)	−0.0076 (9)	−0.0105 (10)
Na5	0.0196 (10)	0.0193 (10)	0.0213 (10)	−0.0013 (8)	−0.0044 (8)	−0.0083 (8)
Na6	0.0196 (11)	0.0406 (14)	0.0390 (13)	0.0043 (10)	−0.0025 (9)	−0.0292 (11)
O1C	0.0109 (15)	0.0103 (16)	0.0102 (15)	0.0030 (12)	−0.0029 (12)	−0.0083 (13)
O2C	0.0134 (13)	0.0141 (14)	0.0093 (13)	−0.0001 (11)	−0.0014 (10)	−0.0088 (11)
O3C	0.0123 (16)	0.0073 (16)	0.0114 (16)	−0.0029 (13)	−0.0031 (12)	−0.0049 (13)
O4C	0.0082 (16)	0.0139 (17)	0.0170 (17)	0.0010 (13)	−0.0028 (13)	−0.0115 (14)
O5C	0.0134 (16)	0.0147 (16)	0.0107 (15)	0.0002 (13)	−0.0019 (12)	−0.0106 (13)
O6C	0.0096 (16)	0.0118 (17)	0.0130 (16)	−0.0011 (13)	−0.0008 (12)	−0.0054 (14)
O7B	0.0179 (18)	0.0164 (18)	0.0179 (18)	−0.0008 (14)	−0.0056 (14)	−0.0083 (15)
O8B	0.0117 (16)	0.0169 (17)	0.0131 (16)	−0.0017 (13)	−0.0007 (12)	−0.0076 (14)
O9B	0.0162 (17)	0.0183 (18)	0.0122 (16)	−0.0005 (14)	−0.0028 (13)	−0.0098 (14)
O10B	0.0210 (18)	0.0127 (17)	0.0143 (17)	−0.0001 (14)	−0.0017 (13)	−0.0079 (14)
O11B	0.0175 (17)	0.0145 (17)	0.0120 (16)	0.0014 (14)	0.0016 (13)	−0.0046 (14)
O12B	0.0171 (17)	0.0152 (17)	0.0147 (17)	0.0039 (14)	−0.0060 (13)	−0.0088 (14)
O13T	0.0180 (19)	0.028 (2)	0.0227 (19)	0.0080 (16)	−0.0045 (14)	−0.0154 (16)
O14T	0.034 (2)	0.0139 (18)	0.0215 (19)	−0.0022 (16)	−0.0071 (16)	−0.0049 (15)
O15T	0.0132 (17)	0.025 (2)	0.0213 (18)	0.0026 (15)	−0.0010 (14)	−0.0142 (16)
O16T	0.0179 (18)	0.0210 (19)	0.0146 (17)	0.0004 (14)	−0.0025 (13)	−0.0125 (15)
O17T	0.0168 (17)	0.0228 (19)	0.0122 (16)	−0.0011 (14)	−0.0023 (13)	−0.0104 (15)
O18T	0.0229 (19)	0.0205 (19)	0.0144 (18)	−0.0061 (15)	−0.0014 (14)	−0.0051 (15)
O19T	0.031 (2)	0.0169 (19)	0.0208 (19)	−0.0033 (15)	−0.0048 (15)	−0.0063 (15)
O20T	0.0149 (18)	0.029 (2)	0.027 (2)	0.0067 (16)	−0.0029 (15)	−0.0146 (17)
O21T	0.0176 (19)	0.029 (2)	0.025 (2)	0.0050 (16)	0.0054 (15)	−0.0127 (17)
O22T	0.033 (2)	0.024 (2)	0.0157 (18)	−0.0011 (16)	−0.0057 (15)	−0.0140 (16)
O23T	0.032 (2)	0.035 (2)	0.0200 (19)	0.0023 (17)	−0.0101 (16)	−0.0176 (17)
O24T	0.030 (2)	0.0184 (19)	0.0189 (19)	−0.0024 (16)	−0.0004 (15)	−0.0012 (16)
O25C	0.0109 (13)	0.0106 (13)	0.0118 (13)	0.0017 (11)	−0.0010 (10)	−0.0095 (11)
O26C	0.0106 (16)	0.0088 (16)	0.0126 (16)	−0.0009 (13)	−0.0011 (13)	−0.0046 (13)
O27C	0.0116 (16)	0.0128 (17)	0.0118 (16)	0.0008 (13)	−0.0036 (12)	−0.0097 (13)
O28C	0.0078 (16)	0.0139 (17)	0.0137 (16)	0.0008 (13)	−0.0028 (12)	−0.0107 (13)
O29C	0.0149 (16)	0.0083 (16)	0.0090 (15)	−0.0016 (13)	−0.0019 (12)	−0.0033 (13)
O30C	0.0146 (16)	0.0170 (16)	0.0071 (14)	0.0015 (13)	−0.0025 (12)	−0.0086 (13)
O31B	0.0172 (18)	0.0169 (18)	0.0187 (18)	−0.0032 (14)	−0.0050 (14)	−0.0098 (15)
O32B	0.0168 (17)	0.0174 (18)	0.0147 (17)	0.0048 (14)	−0.0062 (13)	−0.0092 (14)
O33B	0.0142 (17)	0.0170 (18)	0.0115 (16)	−0.0008 (14)	−0.0005 (12)	−0.0060 (14)
O34B	0.0181 (17)	0.0137 (17)	0.0123 (16)	0.0003 (14)	−0.0036 (13)	−0.0068 (14)
O35B	0.0151 (17)	0.0201 (18)	0.0122 (16)	0.0034 (14)	−0.0029 (13)	−0.0104 (14)
O36B	0.0125 (16)	0.0164 (17)	0.0115 (16)	−0.0040 (13)	0.0004 (12)	−0.0079 (14)
O37T	0.0159 (18)	0.032 (2)	0.0221 (19)	0.0039 (16)	−0.0011 (14)	−0.0178 (17)
O38T	0.0196 (18)	0.0205 (19)	0.0149 (17)	−0.0046 (15)	−0.0007 (14)	−0.0097 (15)
O39T	0.0205 (19)	0.033 (2)	0.030 (2)	0.0133 (17)	−0.0092 (16)	−0.0201 (18)
O40T	0.039 (2)	0.0172 (19)	0.025 (2)	−0.0015 (17)	−0.0113 (17)	−0.0063 (16)
O41T	0.030 (2)	0.0172 (19)	0.0171 (18)	−0.0043 (16)	−0.0003 (15)	−0.0032 (15)
O42T	0.028 (2)	0.026 (2)	0.0211 (19)	0.0054 (16)	−0.0140 (15)	−0.0138 (16)
O43T	0.0244 (19)	0.0236 (19)	0.0180 (18)	0.0016 (15)	−0.0083 (14)	−0.0135 (16)
O44T	0.0156 (18)	0.027 (2)	0.0215 (19)	0.0048 (15)	−0.0002 (14)	−0.0134 (16)
O45T	0.0208 (19)	0.031 (2)	0.025 (2)	0.0141 (16)	−0.0094 (15)	−0.0179 (17)
O46T	0.036 (2)	0.0189 (19)	0.025 (2)	−0.0033 (16)	−0.0065 (16)	−0.0082 (16)
O47T	0.026 (2)	0.0193 (19)	0.0133 (18)	−0.0052 (15)	−0.0020 (14)	−0.0045 (15)
O48T	0.0179 (18)	0.0215 (19)	0.0121 (17)	−0.0015 (14)	−0.0030 (13)	−0.0069 (15)
O1W	0.028 (2)	0.034 (2)	0.019 (2)	−0.0081 (18)	−0.0076 (16)	−0.0033 (17)
O2W	0.021 (2)	0.032 (2)	0.026 (2)	−0.0052 (17)	0.0003 (16)	−0.0175 (18)
O3W	0.027 (2)	0.027 (2)	0.023 (2)	0.0053 (17)	−0.0023 (16)	−0.0141 (17)
O4W	0.045 (3)	0.027 (2)	0.030 (2)	−0.0024 (19)	−0.0093 (19)	−0.0144 (18)
O5W	0.018 (2)	0.044 (3)	0.053 (3)	0.0046 (19)	−0.0056 (19)	−0.039 (2)
O6W	0.0196 (19)	0.021 (2)	0.0224 (19)	−0.0023 (15)	−0.0023 (15)	−0.0101 (16)
O7W	0.022 (2)	0.028 (2)	0.035 (2)	0.0009 (17)	−0.0071 (17)	−0.0165 (18)
O8W	0.029 (2)	0.025 (2)	0.024 (2)	−0.0001 (17)	−0.0029 (16)	−0.0139 (17)
O9W	0.046 (3)	0.039 (3)	0.026 (2)	0.014 (2)	−0.0111 (18)	−0.0095 (19)
O10W	0.047 (3)	0.062 (3)	0.030 (2)	0.006 (2)	−0.003 (2)	−0.015 (2)
O11W	0.0184 (19)	0.032 (2)	0.036 (2)	−0.0039 (17)	−0.0055 (16)	−0.0239 (18)
O12W	0.033 (3)	0.122 (5)	0.047 (3)	−0.013 (3)	0.003 (2)	−0.059 (3)
O13W	0.031 (2)	0.038 (2)	0.029 (2)	0.0038 (18)	−0.0092 (17)	−0.0140 (18)
O14W	0.0219 (19)	0.026 (2)	0.025 (2)	−0.0024 (16)	−0.0042 (15)	−0.0134 (17)
O15W	0.0180 (18)	0.030 (2)	0.0205 (19)	0.0008 (16)	−0.0039 (15)	−0.0124 (17)
O16W	0.029 (3)	0.104 (4)	0.052 (3)	−0.008 (3)	−0.001 (2)	−0.050 (3)
O17W	0.025 (2)	0.025 (2)	0.023 (2)	−0.0075 (17)	−0.0051 (16)	−0.0021 (17)
O18W	0.024 (2)	0.024 (2)	0.028 (2)	−0.0037 (17)	−0.0076 (17)	−0.0068 (18)
O19W	0.022 (2)	0.025 (2)	0.036 (2)	−0.0005 (17)	−0.0053 (17)	−0.0087 (19)
O20W	0.086 (5)	0.045 (3)	0.084 (5)	−0.003 (3)	0.015 (3)	−0.026 (3)
O21W	0.056 (3)	0.109 (5)	0.033 (3)	0.002 (3)	0.007 (2)	−0.024 (3)
O22W	0.051 (3)	0.056 (3)	0.063 (3)	0.013 (2)	−0.022 (3)	−0.041 (3)
O23W	0.033 (2)	0.077 (3)	0.044 (3)	−0.017 (2)	−0.004 (2)	−0.037 (3)
O24W	0.065 (3)	0.042 (3)	0.029 (2)	0.008 (2)	−0.023 (2)	−0.014 (2)
O25W	0.059 (3)	0.052 (3)	0.046 (3)	−0.027 (2)	0.021 (2)	−0.033 (3)
O26W	0.047 (3)	0.050 (3)	0.069 (3)	0.005 (2)	−0.011 (2)	−0.037 (3)
O27W	0.041 (3)	0.038 (3)	0.034 (2)	−0.007 (2)	−0.0026 (19)	−0.015 (2)
O28W	0.024 (2)	0.036 (2)	0.034 (2)	−0.0010 (18)	−0.0077 (18)	−0.0154 (19)
O29W	0.032 (3)	0.036 (3)	0.071 (3)	−0.003 (2)	−0.019 (2)	−0.022 (3)

### Geometric parameters (Å, º)

**Table:**

Mo1—O1C	2.114 (3)	Na4—O10W^v^	2.428 (5)
Mo6—O1C	2.198 (3)	Na4—O13W	2.495 (4)
Mo1—O2C	2.216 (3)	Na4—O11W^ii^	2.534 (4)
Mo2—O2C	2.246 (3)	Na5—O18W^vi^	2.322 (4)
Mo2—O3C	2.245 (3)	Na5—O41T	2.364 (4)
Mo3—O3C	2.336 (3)	Na5—O12W^vii^	2.373 (4)
Mo3—O4C	2.267 (3)	Na5—O39T^iv^	2.406 (4)
Mo4—O4C	2.283 (3)	Na5—O17W	2.419 (4)
Mo4—O5C	2.312 (3)	Na5—O14W^vii^	2.478 (4)
Mo5—O5C	2.280 (3)	Na6—O45T^viii^	2.363 (4)
Mo5—O6C	2.358 (3)	Na6—O16W^vii^	2.386 (5)
Mo6—O6C	2.287 (3)	Na6—O5W	2.387 (5)
Mo7—O25C	2.186 (3)	Na6—O44T	2.390 (4)
Mo12—O25C	2.084 (3)	Na6—O5W^viii^	2.436 (5)
Mo7—O26C	2.297 (3)	Na6—O20W^vii^	2.437 (6)
Mo8—O26C	2.305 (3)	O2C—O30C	2.595 (5)
Mo8—O27C	2.272 (3)	O2C—H2	0.86 (3)
Mo9—O27C	2.302 (3)	O3C—H3	0.86 (3)
Mo9—O28C	2.307 (3)	O4C—H4	0.85 (3)
Mo10—O28C	2.302 (3)	O5C—H5	0.86 (3)
Mo10—O29C	2.196 (3)	O6C—H6	0.86 (3)
Mo11—O29C	2.122 (3)	O7B—H7	0.84 (3)
Mo11—O30C	2.359 (3)	O13T—Na1^i^	2.483 (4)
Mo12—O30C	2.340 (3)	O15T—Na2^i^	2.369 (4)
Mo1—O7B	2.098 (3)	O21T—Na3^iii^	2.379 (4)
Mo6—O7B	2.076 (3)	O26C—H26	0.82 (3)
Mo1—O8B	1.883 (3)	O27C—H27	0.87 (3)
Mo2—O8B	1.963 (3)	O28C—H28	0.86 (3)
Mo2—O9B	1.924 (3)	O30C—H30	0.84 (3)
Mo3—O9B	1.953 (3)	O31B—H31	0.82 (3)
Mo3—O10B	1.927 (3)	O39T—Na5^iv^	2.406 (4)
Mo4—O10B	1.947 (3)	O42T—Na4^iv^	2.394 (4)
Mo4—O11B	1.916 (3)	O45T—Na6^viii^	2.363 (4)
Mo5—O11B	1.935 (3)	O1W—H1A	0.87 (3)
Mo5—O12B	1.947 (3)	O1W—H1B	0.84 (3)
Mo6—O12B	1.906 (3)	O2W—H2A	0.86 (3)
Mo7—O31B	2.072 (3)	O2W—H2B	0.87 (3)
Mo12—O31B	2.090 (3)	O3W—Na3^iii^	2.424 (4)
Mo7—O32B	1.899 (3)	O3W—H3A	0.87 (3)
Mo8—O32B	1.935 (3)	O3W—H3B	0.86 (3)
Mo8—O33B	1.959 (3)	O4W—Na3^ix^	2.299 (4)
Mo9—O33B	1.932 (3)	O4W—Na4^ix^	2.330 (4)
Mo9—O34B	1.925 (3)	O4W—H4A	0.88 (3)
Mo10—O34B	1.961 (3)	O4W—H4B	0.82 (3)
Mo10—O35B	1.988 (3)	O5W—Na6^viii^	2.436 (5)
Mo11—O35B	1.947 (3)	O5W—H5A	0.85 (3)
Mo11—O36B	1.970 (3)	O5W—H5B	0.81 (3)
Mo12—O36B	1.870 (3)	O6W—H6A	0.86 (3)
Pt1—O1C	1.978 (3)	O6W—H6B	0.87 (3)
Pt1—O6C	1.980 (3)	O7W—H7A	0.82 (3)
Pt1—O2C	1.984 (3)	O7W—H7B	0.82 (3)
Pt1—O3C	1.992 (3)	O8W—H8A	0.87 (3)
Pt1—O4C	2.018 (3)	O8W—H8B	0.87 (3)
Pt1—O5C	2.029 (3)	O9W—Na4^ix^	2.395 (4)
Pt1—Mo1	3.1985 (4)	O9W—H9A	0.83 (3)
Pt2—O29C	1.977 (3)	O9W—H9B	0.86 (3)
Pt2—O25C	1.993 (3)	O10W—Na4^x^	2.428 (5)
Pt2—O30C	1.995 (3)	O10W—H10A	0.84 (3)
Pt2—O26C	2.002 (3)	O10W—H10B	0.86 (3)
Pt2—O28C	2.006 (3)	O11W—Na4^ix^	2.534 (4)
Pt2—O27C	2.011 (3)	O11W—H11A	0.85 (3)
Mo1—O15T	1.707 (3)	O11W—H11B	0.85 (3)
Mo1—O16T	1.734 (3)	O12W—Na5^xi^	2.373 (4)
Mo2—O18T	1.696 (3)	O12W—H12A	0.9800
Mo2—O17T	1.729 (3)	O12W—H12B	0.9800
Mo3—O20T	1.698 (3)	O13W—H13A	0.9900
Mo3—O19T	1.713 (3)	O13W—H13B	0.9900
Mo4—O21T	1.697 (3)	O14W—Na5^xi^	2.478 (4)
Mo4—O22T	1.708 (3)	O14W—H14A	0.9799
Mo5—O24T	1.697 (3)	O14W—H14B	0.9801
Mo5—O23T	1.701 (3)	O15W—O11W^xi^	2.738 (5)
Mo6—O13T	1.696 (3)	O15W—O19W	2.738 (5)
Mo6—O14T	1.696 (3)	O15W—H15A	0.83 (3)
Mo7—O39T	1.695 (3)	O15W—H15B	0.86 (3)
Mo7—O40T	1.697 (4)	O16W—Na6^xi^	2.386 (5)
Mo8—O42T	1.696 (3)	O16W—O24W^i^	2.842 (6)
Mo8—O41T	1.696 (3)	O16W—O7B^i^	3.068 (6)
Mo9—O44T	1.688 (3)	O16W—H16A	0.89 (3)
Mo9—O43T	1.719 (3)	O16W—H16B	0.85 (3)
Mo10—O46T	1.693 (3)	O17W—H17A	0.81 (3)
Mo10—O45T	1.701 (3)	O17W—H17B	0.84 (3)
Mo10—Mo11	3.2096 (5)	O18W—Na5^xii^	2.322 (4)
Mo11—O47T	1.699 (3)	O18W—H18A	0.83 (3)
Mo11—O48T	1.735 (3)	O18W—H18B	0.81 (3)
Mo12—O37T	1.694 (3)	O19W—H19A	0.85 (3)
Mo12—O38T	1.750 (3)	O19W—H19B	0.80 (3)
Na1—O24T	2.326 (4)	O20W—Na6^xi^	2.437 (6)
Na1—O6W	2.349 (4)	O20W—H20A	0.88 (3)
Na1—O2W	2.377 (4)	O20W—H20B	0.85 (3)
Na1—O1W	2.380 (4)	O21W—H21A	0.89 (3)
Na1—O19W	2.443 (4)	O21W—H21B	0.86 (3)
Na1—O13T^i^	2.483 (4)	O22W—H22A	0.88 (3)
Na2—O15T^i^	2.369 (4)	O22W—H22B	0.85 (3)
Na2—O7W	2.377 (4)	O23W—H23A	0.87 (3)
Na2—O6W	2.388 (4)	O23W—H23B	0.85 (3)
Na2—O15W	2.393 (4)	O24W—H24A	0.84 (3)
Na2—O8W	2.408 (4)	O24W—H24B	0.89 (3)
Na2—O2W	2.434 (4)	O25W—H25A	0.83 (3)
Na3—O4W^ii^	2.299 (4)	O25W—H25B	0.86 (3)
Na3—O3W	2.347 (4)	O26W—H26A	0.87 (3)
Na3—O20T	2.348 (4)	O26W—H26B	0.87 (3)
Na3—O21T^iii^	2.379 (4)	O27W—H27A	0.86 (3)
Na3—O13W	2.390 (4)	O27W—H27B	0.87 (3)
Na3—O3W^iii^	2.424 (4)	O28W—H28A	0.86 (3)
Na3—Na3^iii^	3.379 (4)	O28W—H28B	0.86 (3)
Na4—O4W^ii^	2.330 (4)	O29W—H29A	0.86 (3)
Na4—O42T^iv^	2.394 (4)	O29W—H29B	0.84 (3)
Na4—O9W^ii^	2.395 (4)		
			
Mo1—O1C—Mo6	104.42 (13)	O46T—Mo10—O35B	101.20 (15)
Mo1—O2C—Mo2	93.27 (12)	O45T—Mo10—O35B	94.06 (15)
Mo2—O3C—Mo3	92.61 (11)	O34B—Mo10—O35B	152.77 (13)
Mo3—O4C—Mo4	94.36 (12)	O46T—Mo10—O29C	93.58 (15)
Mo5—O5C—Mo4	93.05 (11)	O45T—Mo10—O29C	157.99 (15)
Mo6—O6C—Mo5	91.98 (11)	O34B—Mo10—O29C	86.01 (12)
Mo6—O7B—Mo1	109.51 (15)	O35B—Mo10—O29C	73.14 (12)
Mo1—O8B—Mo2	115.01 (15)	O46T—Mo10—O28C	161.08 (15)
Mo2—O9B—Mo3	117.38 (16)	O45T—Mo10—O28C	90.14 (15)
Mo3—O10B—Mo4	118.97 (16)	O34B—Mo10—O28C	70.72 (12)
Mo4—O11B—Mo5	119.86 (16)	O35B—Mo10—O28C	85.87 (12)
Mo6—O12B—Mo5	120.24 (16)	O29C—Mo10—O28C	71.49 (11)
Mo12—O25C—Mo7	104.38 (13)	O46T—Mo10—Mo11	88.46 (12)
Mo7—O26C—Mo8	92.23 (11)	O45T—Mo10—Mo11	128.96 (12)
Mo8—O27C—Mo9	93.67 (11)	O34B—Mo10—Mo11	127.13 (9)
Mo10—O28C—Mo9	93.85 (11)	O35B—Mo10—Mo11	34.92 (9)
Mo11—O29C—Mo10	96.01 (12)	O29C—Mo10—Mo11	41.12 (8)
Mo12—O30C—Mo11	90.63 (11)	O28C—Mo10—Mo11	87.55 (8)
Mo7—O31B—Mo12	108.35 (15)	O47T—Mo11—O48T	105.57 (15)
Mo7—O32B—Mo8	119.84 (16)	O47T—Mo11—O35B	101.45 (15)
Mo9—O33B—Mo8	118.01 (15)	O48T—Mo11—O35B	95.26 (14)
Mo9—O34B—Mo10	120.01 (16)	O47T—Mo11—O36B	97.92 (15)
Mo11—O35B—Mo10	109.31 (15)	O48T—Mo11—O36B	97.46 (14)
Mo12—O36B—Mo11	120.93 (15)	O35B—Mo11—O36B	153.01 (13)
O1C—Pt1—O6C	82.75 (13)	O47T—Mo11—O29C	97.13 (14)
O1C—Pt1—O2C	83.08 (12)	O48T—Mo11—O29C	156.88 (14)
O6C—Pt1—O2C	98.53 (13)	O35B—Mo11—O29C	75.62 (12)
O1C—Pt1—O3C	98.76 (13)	O36B—Mo11—O29C	83.51 (12)
O6C—Pt1—O3C	177.87 (12)	O47T—Mo11—O30C	165.79 (14)
O2C—Pt1—O3C	83.17 (13)	O48T—Mo11—O30C	86.13 (14)
O1C—Pt1—O4C	178.01 (12)	O35B—Mo11—O30C	85.13 (12)
O6C—Pt1—O4C	95.27 (13)	O36B—Mo11—O30C	72.13 (12)
O2C—Pt1—O4C	97.15 (13)	O29C—Mo11—O30C	72.11 (11)
O3C—Pt1—O4C	83.22 (13)	O47T—Mo11—Mo10	90.36 (11)
O1C—Pt1—O5C	97.43 (12)	O48T—Mo11—Mo10	131.02 (11)
O6C—Pt1—O5C	82.98 (13)	O35B—Mo11—Mo10	35.77 (9)
O2C—Pt1—O5C	178.47 (12)	O36B—Mo11—Mo10	126.38 (9)
O3C—Pt1—O5C	95.32 (13)	O29C—Mo11—Mo10	42.87 (8)
O4C—Pt1—O5C	82.39 (13)	O30C—Mo11—Mo10	87.77 (7)
O1C—Pt1—Mo1	40.13 (8)	O37T—Mo12—O38T	105.28 (16)
O6C—Pt1—Mo1	93.90 (9)	O37T—Mo12—O36B	102.09 (16)
O2C—Pt1—Mo1	43.18 (9)	O38T—Mo12—O36B	102.12 (15)
O3C—Pt1—Mo1	88.22 (9)	O37T—Mo12—O25C	95.72 (14)
O4C—Pt1—Mo1	140.25 (9)	O38T—Mo12—O25C	152.97 (13)
O5C—Pt1—Mo1	137.18 (9)	O36B—Mo12—O25C	89.63 (12)
O29C—Pt2—O25C	97.99 (13)	O37T—Mo12—O31B	97.63 (15)
O29C—Pt2—O30C	83.50 (13)	O38T—Mo12—O31B	88.31 (14)
O25C—Pt2—O30C	83.64 (12)	O36B—Mo12—O31B	154.25 (13)
O29C—Pt2—O26C	177.72 (12)	O25C—Mo12—O31B	71.95 (12)
O25C—Pt2—O26C	81.67 (12)	O37T—Mo12—O30C	168.56 (14)
O30C—Pt2—O26C	98.68 (13)	O38T—Mo12—O30C	86.14 (14)
O29C—Pt2—O28C	82.62 (13)	O36B—Mo12—O30C	74.22 (12)
O25C—Pt2—O28C	178.26 (11)	O25C—Mo12—O30C	73.62 (11)
O30C—Pt2—O28C	98.06 (13)	O31B—Mo12—O30C	83.21 (12)
O26C—Pt2—O28C	97.66 (13)	O24T—Na1—O6W	169.39 (16)
O29C—Pt2—O27C	95.84 (13)	O24T—Na1—O2W	91.74 (14)
O25C—Pt2—O27C	96.06 (12)	O6W—Na1—O2W	90.55 (14)
O30C—Pt2—O27C	179.23 (13)	O24T—Na1—O1W	87.12 (14)
O26C—Pt2—O27C	81.97 (13)	O6W—Na1—O1W	91.11 (14)
O28C—Pt2—O27C	82.25 (12)	O2W—Na1—O1W	176.82 (17)
O15T—Mo1—O16T	105.41 (15)	O24T—Na1—O19W	99.77 (14)
O15T—Mo1—O8B	100.56 (15)	O6W—Na1—O19W	90.84 (14)
O16T—Mo1—O8B	101.85 (15)	O2W—Na1—O19W	79.87 (14)
O15T—Mo1—O7B	95.79 (15)	O1W—Na1—O19W	97.40 (15)
O16T—Mo1—O7B	88.94 (15)	O24T—Na1—O13T^i^	82.35 (14)
O8B—Mo1—O7B	157.14 (13)	O6W—Na1—O13T^i^	87.56 (14)
O15T—Mo1—O1C	91.61 (14)	O2W—Na1—O13T^i^	84.58 (13)
O16T—Mo1—O1C	155.61 (13)	O1W—Na1—O13T^i^	98.20 (14)
O8B—Mo1—O1C	91.78 (13)	O19W—Na1—O13T^i^	164.35 (15)
O7B—Mo1—O1C	71.77 (12)	O15T^i^—Na2—O7W	91.66 (14)
O15T—Mo1—O2C	165.40 (14)	O15T^i^—Na2—O6W	95.56 (13)
O16T—Mo1—O2C	89.18 (14)	O7W—Na2—O6W	91.55 (14)
O8B—Mo1—O2C	75.40 (12)	O15T^i^—Na2—O15W	171.51 (15)
O7B—Mo1—O2C	84.78 (13)	O7W—Na2—O15W	96.52 (14)
O1C—Mo1—O2C	74.66 (11)	O6W—Na2—O15W	82.00 (13)
O15T—Mo1—Pt1	128.03 (11)	O15T^i^—Na2—O8W	92.83 (13)
O16T—Mo1—Pt1	125.74 (11)	O7W—Na2—O8W	90.84 (14)
O8B—Mo1—Pt1	79.21 (9)	O6W—Na2—O8W	171.21 (15)
O7B—Mo1—Pt1	78.14 (9)	O15W—Na2—O8W	89.33 (13)
O1C—Mo1—Pt1	37.08 (8)	O15T^i^—Na2—O2W	91.24 (14)
O2C—Mo1—Pt1	37.78 (8)	O7W—Na2—O2W	177.09 (15)
O18T—Mo2—O17T	105.58 (15)	O6W—Na2—O2W	88.28 (14)
O18T—Mo2—O9B	100.97 (15)	O15W—Na2—O2W	80.57 (14)
O17T—Mo2—O9B	99.27 (14)	O8W—Na2—O2W	88.91 (14)
O18T—Mo2—O8B	95.89 (15)	O4W^ii^—Na3—O3W	174.07 (17)
O17T—Mo2—O8B	99.65 (14)	O4W^ii^—Na3—O20T	87.99 (15)
O9B—Mo2—O8B	150.17 (13)	O3W—Na3—O20T	87.55 (14)
O18T—Mo2—O3C	88.95 (14)	O4W^ii^—Na3—O21T^iii^	103.88 (16)
O17T—Mo2—O3C	164.86 (13)	O3W—Na3—O21T^iii^	80.97 (14)
O9B—Mo2—O3C	73.24 (12)	O20T—Na3—O21T^iii^	166.84 (15)
O8B—Mo2—O3C	82.72 (12)	O4W^ii^—Na3—O13W	86.52 (14)
O18T—Mo2—O2C	158.87 (14)	O3W—Na3—O13W	96.38 (14)
O17T—Mo2—O2C	94.25 (14)	O20T—Na3—O13W	78.05 (13)
O9B—Mo2—O2C	82.57 (13)	O21T^iii^—Na3—O13W	96.75 (14)
O8B—Mo2—O2C	73.23 (12)	O4W^ii^—Na3—O3W^iii^	87.20 (14)
O3C—Mo2—O2C	71.99 (11)	O3W—Na3—O3W^iii^	89.82 (14)
O20T—Mo3—O19T	106.27 (17)	O20T—Na3—O3W^iii^	101.04 (14)
O20T—Mo3—O10B	101.86 (15)	O21T^iii^—Na3—O3W^iii^	85.43 (14)
O19T—Mo3—O10B	99.45 (15)	O13W—Na3—O3W^iii^	173.68 (16)
O20T—Mo3—O9B	97.79 (15)	O4W^ii^—Na4—O42T^iv^	156.09 (16)
O19T—Mo3—O9B	100.12 (15)	O4W^ii^—Na4—O9W^ii^	92.04 (16)
O10B—Mo3—O9B	147.01 (13)	O42T^iv^—Na4—O9W^ii^	102.56 (15)
O20T—Mo3—O4C	91.34 (15)	O4W^ii^—Na4—O10W^v^	83.94 (16)
O19T—Mo3—O4C	161.54 (14)	O42T^iv^—Na4—O10W^v^	85.19 (15)
O10B—Mo3—O4C	70.87 (12)	O9W^ii^—Na4—O10W^v^	167.83 (18)
O9B—Mo3—O4C	82.51 (13)	O4W^ii^—Na4—O13W	83.45 (14)
O20T—Mo3—O3C	159.41 (15)	O42T^iv^—Na4—O13W	78.16 (13)
O19T—Mo3—O3C	92.80 (14)	O9W^ii^—Na4—O13W	88.64 (15)
O10B—Mo3—O3C	82.12 (12)	O10W^v^—Na4—O13W	102.26 (16)
O9B—Mo3—O3C	70.67 (12)	O4W^ii^—Na4—O11W^ii^	123.93 (15)
O4C—Mo3—O3C	70.67 (11)	O42T^iv^—Na4—O11W^ii^	74.82 (13)
O21T—Mo4—O22T	106.64 (17)	O9W^ii^—Na4—O11W^ii^	92.35 (14)
O21T—Mo4—O11B	97.82 (16)	O10W^v^—Na4—O11W^ii^	80.51 (15)
O22T—Mo4—O11B	102.09 (15)	O13W—Na4—O11W^ii^	152.51 (14)
O21T—Mo4—O10B	101.48 (15)	O18W^vi^—Na5—O41T	169.60 (16)
O22T—Mo4—O10B	96.38 (15)	O18W^vi^—Na5—O12W^vii^	106.16 (18)
O11B—Mo4—O10B	148.14 (13)	O41T—Na5—O12W^vii^	81.20 (16)
O21T—Mo4—O4C	93.57 (15)	O18W^vi^—Na5—O39T^iv^	92.76 (15)
O22T—Mo4—O4C	157.85 (14)	O41T—Na5—O39T^iv^	79.54 (14)
O11B—Mo4—O4C	83.58 (13)	O12W^vii^—Na5—O39T^iv^	91.39 (15)
O10B—Mo4—O4C	70.20 (12)	O18W^vi^—Na5—O17W	93.24 (15)
O21T—Mo4—O5C	161.20 (15)	O41T—Na5—O17W	80.61 (13)
O22T—Mo4—O5C	90.59 (14)	O12W^vii^—Na5—O17W	159.22 (19)
O11B—Mo4—O5C	70.61 (12)	O39T^iv^—Na5—O17W	95.08 (14)
O10B—Mo4—O5C	83.57 (12)	O18W^vi^—Na5—O14W^vii^	97.57 (14)
O4C—Mo4—O5C	70.91 (11)	O41T—Na5—O14W^vii^	90.15 (14)
O24T—Mo5—O23T	106.92 (18)	O12W^vii^—Na5—O14W^vii^	86.13 (15)
O24T—Mo5—O11B	97.36 (16)	O39T^iv^—Na5—O14W^vii^	169.66 (15)
O23T—Mo5—O11B	101.02 (16)	O17W—Na5—O14W^vii^	83.98 (13)
O24T—Mo5—O12B	101.20 (15)	O45T^viii^—Na6—O16W^vii^	84.88 (15)
O23T—Mo5—O12B	99.38 (15)	O45T^viii^—Na6—O5W	94.17 (14)
O11B—Mo5—O12B	146.97 (13)	O16W^vii^—Na6—O5W	177.0 (2)
O24T—Mo5—O5C	157.91 (15)	O45T^viii^—Na6—O44T	171.00 (16)
O23T—Mo5—O5C	93.95 (15)	O16W^vii^—Na6—O44T	97.15 (15)
O11B—Mo5—O5C	71.01 (12)	O5W—Na6—O44T	83.36 (14)
O12B—Mo5—O5C	81.93 (12)	O45T^viii^—Na6—O5W^viii^	86.65 (14)
O24T—Mo5—O6C	90.40 (15)	O16W^vii^—Na6—O5W^viii^	93.72 (18)
O23T—Mo5—O6C	161.60 (15)	O5W—Na6—O5W^viii^	83.36 (15)
O11B—Mo5—O6C	82.37 (12)	O44T—Na6—O5W^viii^	84.47 (14)
O12B—Mo5—O6C	70.49 (12)	O45T^viii^—Na6—O20W^vii^	101.56 (18)
O5C—Mo5—O6C	69.85 (11)	O16W^vii^—Na6—O20W^vii^	81.7 (2)
O13T—Mo6—O14T	107.19 (18)	O5W—Na6—O20W^vii^	101.3 (2)
O13T—Mo6—O12B	100.66 (15)	O44T—Na6—O20W^vii^	87.42 (18)
O14T—Mo6—O12B	103.65 (15)	O5W^viii^—Na6—O20W^vii^	170.2 (2)
O13T—Mo6—O7B	98.18 (15)	Pt1—O1C—Mo1	102.79 (13)
O14T—Mo6—O7B	90.40 (15)	Pt1—O1C—Mo6	104.47 (13)
O12B—Mo6—O7B	151.86 (13)	Pt1—O2C—Mo1	99.04 (13)
O13T—Mo6—O1C	93.47 (15)	Pt1—O2C—Mo2	102.52 (14)
O14T—Mo6—O1C	153.85 (15)	Pt1—O2C—O30C	116.35 (16)
O12B—Mo6—O1C	87.60 (12)	Pt1—O3C—Mo2	102.31 (13)
O7B—Mo6—O1C	70.51 (12)	Pt1—O3C—Mo3	102.23 (13)
O13T—Mo6—O6C	163.39 (15)	Pt1—O4C—Mo3	103.81 (13)
O14T—Mo6—O6C	89.30 (15)	Pt1—O4C—Mo4	104.05 (13)
O12B—Mo6—O6C	72.79 (12)	Pt1—O5C—Mo5	104.17 (14)
O7B—Mo6—O6C	83.29 (12)	Pt1—O5C—Mo4	102.66 (13)
O1C—Mo6—O6C	71.32 (11)	Pt1—O6C—Mo6	101.23 (13)
O39T—Mo7—O40T	107.31 (19)	Pt1—O6C—Mo5	103.00 (13)
O39T—Mo7—O32B	101.06 (15)	Pt2—O25C—Mo12	105.47 (13)
O40T—Mo7—O32B	104.07 (15)	Pt2—O25C—Mo7	105.62 (13)
O39T—Mo7—O31B	97.15 (15)	Mo1—O2C—O30C	122.10 (17)
O40T—Mo7—O31B	89.78 (15)	Mo2—O2C—O30C	119.13 (16)
O32B—Mo7—O31B	152.51 (13)	Pt2—O26C—Mo7	101.33 (13)
O39T—Mo7—O25C	93.78 (15)	Pt2—O26C—Mo8	103.45 (13)
O40T—Mo7—O25C	152.78 (15)	Pt2—O27C—Mo8	104.35 (13)
O32B—Mo7—O25C	88.06 (13)	Pt2—O27C—Mo9	103.89 (13)
O31B—Mo7—O25C	70.25 (12)	Pt2—O28C—Mo10	100.58 (13)
O39T—Mo7—O26C	163.50 (15)	Pt2—O28C—Mo9	103.89 (13)
O40T—Mo7—O26C	89.09 (15)	Pt2—O29C—Mo11	106.63 (14)
O32B—Mo7—O26C	72.35 (12)	Pt2—O29C—Mo10	105.30 (13)
O31B—Mo7—O26C	84.45 (12)	Pt2—O30C—Mo12	96.59 (13)
O25C—Mo7—O26C	71.22 (11)	Pt2—O30C—Mo11	97.72 (13)
O42T—Mo8—O41T	107.11 (17)	Pt2—O30C—O2C	117.78 (16)
O42T—Mo8—O32B	98.16 (15)	Mo12—O30C—O2C	124.51 (17)
O41T—Mo8—O32B	102.73 (15)	Mo11—O30C—O2C	122.82 (16)
O42T—Mo8—O33B	99.95 (15)	H1A—O1W—H1B	109 (4)
O41T—Mo8—O33B	96.08 (15)	H2A—O2W—H2B	104 (3)
O32B—Mo8—O33B	148.67 (13)	H3A—O3W—H3B	110 (4)
O42T—Mo8—O27C	93.34 (14)	H4A—O4W—H4B	110 (4)
O41T—Mo8—O27C	157.72 (15)	H5A—O5W—H5B	111 (4)
O32B—Mo8—O27C	82.48 (12)	H6A—O6W—H6B	107 (3)
O33B—Mo8—O27C	71.16 (12)	H7A—O7W—H7B	112 (4)
O42T—Mo8—O26C	161.29 (14)	H8A—O8W—H8B	105 (3)
O41T—Mo8—O26C	90.66 (15)	H9A—O9W—H9B	113 (4)
O32B—Mo8—O26C	71.58 (12)	H10A—O10W—H10B	112 (4)
O33B—Mo8—O26C	83.51 (12)	H11A—O11W—H11B	111 (4)
O27C—Mo8—O26C	70.22 (11)	H12A—O12W—H12B	109.5
O44T—Mo9—O43T	105.42 (16)	H13A—O13W—H13B	110.8
O44T—Mo9—O34B	101.22 (15)	H14A—O14W—H14B	109.5
O43T—Mo9—O34B	97.29 (15)	H15A—O15W—H15B	115 (4)
O44T—Mo9—O33B	98.74 (15)	H16A—O16W—H16B	105 (4)
O43T—Mo9—O33B	101.57 (15)	H17A—O17W—H17B	115 (4)
O34B—Mo9—O33B	147.68 (13)	H18A—O18W—H18B	112 (4)
O44T—Mo9—O27C	159.33 (14)	H19A—O19W—H19B	112 (4)
O43T—Mo9—O27C	94.36 (14)	H20A—O20W—H20B	111 (5)
O34B—Mo9—O27C	81.75 (12)	H21A—O21W—H21B	105 (4)
O33B—Mo9—O27C	70.92 (12)	H22A—O22W—H22B	108 (4)
O44T—Mo9—O28C	91.45 (14)	H23A—O23W—H23B	106 (4)
O43T—Mo9—O28C	161.38 (14)	H24A—O24W—H24B	108 (4)
O34B—Mo9—O28C	71.21 (12)	H25A—O25W—H25B	111 (4)
O33B—Mo9—O28C	83.19 (12)	H26A—O26W—H26B	107 (4)
O27C—Mo9—O28C	69.96 (11)	H27A—O27W—H27B	109 (4)
O46T—Mo10—O45T	106.66 (18)	H28A—O28W—H28B	107 (4)
O46T—Mo10—O34B	97.33 (15)	H29A—O29W—H29B	110 (4)
O45T—Mo10—O34B	99.56 (15)		

Symmetry codes: (i) −*x*+1, −*y*+1, −*z*+1; (ii) *x*, *y*+1, *z*; (iii) −*x*, −*y*+2, −*z*+1; (iv) −*x*, −*y*+2, −*z*; (v) *x*−1, *y*+1, *z*; (vi) *x*, *y*+1, *z*−1; (vii) *x*, *y*, *z*−1; (viii) −*x*+1, −*y*+1, −*z*; (ix) *x*, *y*−1, *z*; (x) *x*+1, *y*−1, *z*; (xi) *x*, *y*, *z*+1; (xii) *x*, *y*−1, *z*+1.

### Hydrogen-bond geometry (Å, º)

**Table:**

*D*—H···*A*	*D*—H	H···*A*	*D*···*A*	*D*—H···*A*
O2*C*—H2···O30*C*	0.86 (3)	1.84 (6)	2.595 (5)	145 (9)
O3*C*—H3···O14*W*^xiii^	0.86 (3)	1.74 (3)	2.586 (5)	164 (4)
O4*C*—H4···O38*T*	0.85 (3)	1.72 (3)	2.576 (4)	178 (5)
O5*C*—H5···O29*W*	0.86 (3)	1.79 (3)	2.595 (5)	156 (5)
O6*C*—H6···O48*T*	0.86 (3)	1.72 (3)	2.569 (4)	171 (5)
O7*B*—H7···O35*B*	0.84 (3)	1.94 (3)	2.785 (5)	175 (5)
O26*C*—H26···O17*T*	0.82 (3)	1.73 (3)	2.556 (4)	178 (5)
O27*C*—H27···O15*W*^xiv^	0.87 (3)	1.70 (3)	2.548 (5)	164 (5)
O28*C*—H28···O16*T*	0.86 (3)	1.73 (3)	2.575 (4)	166 (5)
O30*C*—H30···O2*C*	0.84 (3)	1.76 (3)	2.595 (5)	172 (9)
O31*B*—H31···O9*B*	0.82 (3)	1.95 (3)	2.763 (4)	171 (5)
O1*W*—H1*A*···O36*B*^xiv^	0.87 (3)	1.96 (3)	2.830 (5)	173 (5)
O1*W*—H1*B*···O48*T*	0.84 (3)	2.22 (3)	3.023 (5)	161 (5)
O2*W*—H2*A*···O16*W*	0.86 (3)	1.87 (3)	2.731 (6)	175 (5)
O2*W*—H2*B*···O43*T*^xi^	0.87 (3)	2.17 (3)	3.031 (5)	169 (5)
O3*W*—H3*A*···O22*W*	0.87 (3)	2.10 (3)	2.969 (6)	175 (5)
O3*W*—H3*B*···O38*T*	0.86 (3)	2.13 (3)	2.980 (5)	176 (5)
O4*W*—H4*A*···O26*W*	0.88 (3)	1.98 (3)	2.857 (6)	175 (5)
O4*W*—H4*B*···O25*W*	0.82 (3)	1.99 (3)	2.806 (6)	176 (6)
O5*W*—H5*A*···O16*T*	0.85 (3)	2.08 (3)	2.930 (5)	178 (6)
O6*W*—H6*A*···O25*C*^xiv^	0.86 (3)	2.04 (3)	2.880 (5)	167 (5)
O6*W*—H6*B*···O18*W*	0.87 (3)	1.97 (3)	2.831 (6)	173 (5)
O7*W*—H7*A*···O17*W*^xii^	0.82 (3)	1.98 (3)	2.804 (5)	175 (6)
O7*W*—H7*B*···O32*B*^xiv^	0.82 (3)	2.02 (3)	2.842 (5)	174 (5)
O8*W*—H8*A*···O34*B*^xi^	0.87 (3)	2.21 (3)	3.064 (5)	168 (5)
O8*W*—H8*B*···O9*W*^xi^	0.87 (3)	1.89 (3)	2.750 (6)	169 (5)
O9*W*—H9*A*···O19*T*^ix^	0.83 (3)	2.22 (3)	3.046 (5)	178 (5)
O9*W*—H9*B*···O7*W*^vii^	0.86 (3)	1.91 (3)	2.720 (5)	156 (6)
O10*W*—H10*A*···O25*W*^xv^	0.84 (3)	2.23 (4)	2.924 (7)	140 (5)
O10*W*—H10*B*···O21*W*^i^	0.86 (3)	2.02 (3)	2.874 (7)	174 (7)
O11*W*—H11*A*···O34*B*	0.85 (3)	1.93 (3)	2.723 (5)	155 (5)
O11*W*—H11*B*···O43*T*^xvi^	0.85 (3)	2.08 (3)	2.867 (5)	154 (5)
O12*W*—H12*A*···O22*T*	0.98	2.25	2.904 (5)	123
O12*W*—H12*A*···O21*W*	0.98	2.31	3.141 (8)	142
O13*W*—H13*A*···O12*W*^iii^	0.99	1.80	2.766 (6)	164
O13*W*—H13*B*···O31*B*	0.99	2.53	3.396 (5)	146
O14*W*—H14*A*···O27*W*^xi^	0.98	1.76	2.737 (6)	177
O14*W*—H14*B*···O23*W*	0.98	1.96	2.796 (6)	142
O15*W*—H15*A*···O19*W*	0.83 (3)	1.97 (3)	2.738 (5)	154 (5)
O16*W*—H16*A*···O20*W*	0.89 (3)	2.45 (6)	3.156 (7)	137 (7)
O16*W*—H16*B*···O24*W*^i^	0.85 (3)	2.17 (6)	2.842 (6)	136 (6)
O17*W*—H17*A*···O8*B*^xvii^	0.81 (3)	1.98 (3)	2.790 (5)	173 (5)
O17*W*—H17*B*···O17*T*	0.84 (3)	2.22 (3)	3.027 (5)	160 (5)
O18*W*—H18*A*···O28*W*	0.83 (3)	2.18 (4)	2.907 (5)	146 (5)
O18*W*—H18*B*···O1*C*^i^	0.81 (3)	1.99 (3)	2.798 (5)	176 (5)
O19*W*—H19*A*···O29*C*^xiv^	0.85 (3)	2.01 (3)	2.842 (5)	164 (5)
O19*W*—H19*B*···O10*W*^i^	0.80 (3)	2.14 (3)	2.920 (6)	164 (6)
O20*W*—H20*B*···O23*W*	0.85 (3)	2.46 (7)	3.121 (8)	135 (8)
O21*W*—H21*A*···O23*T*	0.89 (3)	2.24 (4)	3.064 (6)	155 (7)
O21*W*—H21*B*···O33*B*^xi^	0.86 (3)	2.17 (3)	2.972 (5)	155 (6)
O22*W*—H22*A*···O28*W*^xiv^	0.88 (3)	2.28 (5)	3.007 (6)	140 (5)
O22*W*—H22*B*···O26*W*^xiv^	0.85 (3)	1.96 (3)	2.805 (7)	169 (6)
O23*W*—H23*A*···O22*T*	0.87 (3)	2.30 (5)	2.970 (6)	134 (6)
O23*W*—H23*B*···O10*B*^xiii^	0.85 (3)	1.95 (3)	2.775 (5)	162 (7)
O24*W*—H24*A*···O28*W*	0.84 (3)	2.02 (3)	2.854 (6)	173 (6)
O24*W*—H24*B*···O35*B*	0.89 (3)	2.05 (3)	2.911 (5)	163 (5)
O25*W*—H25*A*···O38*T*^xiv^	0.83 (3)	2.52 (6)	3.119 (6)	130 (7)
O25*W*—H25*B*···O47*T*	0.86 (3)	2.01 (3)	2.834 (5)	161 (7)
O26*W*—H26*A*···O24*W*	0.87 (3)	1.93 (5)	2.723 (6)	150 (7)
O26*W*—H26*B*···O19*T*^ix^	0.87 (3)	2.23 (4)	2.920 (5)	135 (5)
O27*W*—H27*A*···O18*T*^xvii^	0.86 (3)	2.33 (5)	2.945 (5)	129 (5)
O27*W*—H27*B*···O33*B*	0.87 (3)	2.10 (4)	2.846 (5)	144 (5)
O28*W*—H28*A*···O12*B*^i^	0.86 (3)	1.93 (3)	2.775 (5)	168 (6)
O28*W*—H28*B*···O1*W*	0.86 (3)	1.96 (3)	2.817 (6)	171 (6)
O29*W*—H29*A*···O22*T*^xiii^	0.86 (3)	2.26 (5)	2.895 (5)	131 (5)
O29*W*—H29*B*···O22*W*^xv^	0.84 (3)	2.03 (3)	2.844 (6)	165 (7)

Symmetry codes: (i) −*x*+1, −*y*+1, −*z*+1; (iii) −*x*, −*y*+2, −*z*+1; (vii) *x*, *y*, *z*−1; (ix) *x*, *y*−1, *z*; (xi) *x*, *y*, *z*+1; (xii) *x*, *y*−1, *z*+1; (xiii) −*x*+1, −*y*+2, −*z*+1; (xiv) −*x*, −*y*+1, −*z*+1; (xv) *x*+1, *y*, *z*; (xvi) −*x*, −*y*+1, −*z*; (xvii) −*x*+1, −*y*+2, −*z*.
